# Endothelial LATS2 is a suppressor of bone marrow fibrosis

**DOI:** 10.1038/s44161-024-00508-x

**Published:** 2024-07-29

**Authors:** Kishor K. Sivaraj, Paul-Georg Majev, Backialakshmi Dharmalingam, Silke Schröder, Bella Banjanin, Martin Stehling, Dagmar Zeuschner, Alfred Nordheim, Rebekka K. Schneider, Ralf H. Adams

**Affiliations:** 1 Department of Tissue Morphogenesis, Max Planck Institute for Molecular Biomedicine, Münster, Germany; 2 Department of Developmental Biology, Erasmus University Medical Center, Rotterdam, The Netherlands; 3 Flow Cytometry Unit, Max Planck Institute for Molecular Biomedicine, Münster, Germany; 4 Electron Microscopy Unit, Max Planck Institute for Molecular Biomedicine, Münster, Germany; 5 Department of Molecular Biology, Interfaculty Institute for Cell Biology, University of Tübingen, Tübingen, Germany; 6 Leibniz Institute on Aging – Fritz Lipmann Institute, Jena, Germany; 7 Oncode Institute, Erasmus University Medical Center, Rotterdam, The Netherlands; 8 Institute for Cell and Tumor Biology, Rheinisch-Westfälische Technische Hochschule (RWTH) Aachen University, Aachen, Germany

**Keywords:** Cardiovascular biology, Calcification

## Abstract

Myelofibrosis and osteosclerosis are fibrotic diseases disrupting bone marrow function that occur in various leukemias but also in response to non-malignant alterations in hematopoietic cells. Here we show that endothelial cell–specific inactivation of the *Lats2* gene, encoding Hippo kinase large tumor suppressor kinase 2, or overexpression of the downstream effector YAP1 induce myofibroblast formation and lead to extensive fibrosis and osteosclerosis, which impair bone marrow function and cause extramedullary hematopoiesis in the spleen. Mechanistically, loss of LATS2 induces endothelial-to-mesenchymal transition, resulting in increased expression of extracellular matrix and secreted signaling molecules. Changes in endothelial cells involve increased expression of serum response factor target genes, and, strikingly, major aspects of the LATS2 mutant phenotype are rescued by inactivation of the *Srf* gene. These findings identify the endothelium as a driver of bone marrow fibrosis, which improves understanding of myelofibrotic and osteosclerotic diseases, for which drug therapies are currently lacking.

## Abstract (web summary)

Sivaraj, Majev et al. demonstrate that the inactivation of Lats2 in endothelial cells triggers the upregulation of serum response factor and endothelial-to-mesenchymal transition, leading to myofibroblast formation, bone marrow fibrosis, osteosclerosis, impaired bone marrow function and extramedullary hematopoiesis.

## Main

Fibrosis is associated with tissue injury, chronic inflammation and numerous other pathologies. In bone, fibrosis occurs in a variety of malignant and non-malignant disease conditions but also after acute or chronic exposure to radiation^1^. In certain groups of rare blood cancers, namely in primary myelofibrosis and in myelodysplastic syndromes, bone marrow (BM) fibrosis is a risk factor and most prevalent in patients with advanced disease and poor prognosis^2^. Excessive deposition of extracellular matrix (ECM) and tissue scarring contribute to impaired BM function, resulting in extramedullary hematopoiesis (EMH) and splenomegaly. Advanced bone fibrosis in human patients, but also in mouse models, can be accompanied by osteosclerosis, which manifests as irregular thickening of trabecular bone and abnormally elevated bone density^3,4^.

Bone marrow mesenchymal stromal cells (BMSCs), especially cells expressing Leptin receptor (LepR) or the zinc finger protein Gli1, were previously shown to contribute to osteogenesis^5–7^. These BMSC populations are also critical mediators of myelofibrosis and osteosclerosis^8–10^. Expression of tumor necrosis factor α and JAK-STAT signaling were shown to be increased in pre-fibrotic BMSCs, whereas strong upregulation of TGFβ signaling is predominant in overt fibrosis^11^. Elevated numbers and aberrant properties of megakaryocytes are also associated with myeloproliferation, BM fibrosis and destruction of the hematopoietic microenvironment^12^. Other alterations affecting hematopoietic cells, such as hypomorphic mutations in the transcription factor GATA1 or high expression of the glycoprotein hormone thrombopoietin (ThPO), cause myelofibrosis in mice^13^.

Although the role of endothelial cells (ECs) in myelofibrosis remains little understood, it was previously shown that ECs of small vessels in BM and spleen of the JAK2-V617F knock-in mouse model, but also in patients with primary myelofibrosis, acquire the expression of mesenchymal markers, which is a hallmark of a process known as endothelial-to-mesenchymal transition (EndMT)^14^. ECs play critical roles in normal osteogenesis and hematopoiesis^15,16^. In the metaphysis, specialized capillary ECs, termed type H because of high expression of the cell surface proteins CD31/Pecam1 and endomucin (EMCN), provide growth factor signals acting on perivascular osteoprogenitors and other osteoblast (OB) lineage cells expressing the transcription factors Runx2 and Osterix (OSX)^16^. The behavior and function of ECs is controlled by external growth factor signals but also by fluid shear stress, matrix stiffness and other mechanical signals^17^. The transcriptional coactivators YAP1 and TAZ, which are effectors in the Hippo signaling pathway, are capable of integrating a range of upstream signals to control cell proliferation, differentiation and tissue growth^18^. YAP1/TAZ interact in the nucleus with DNA-binding transcription factors, such as the TEA domain family members TEAD1–4, and thereby regulate gene expression^19^. Activation of the Hippo signaling cascade and large tumor suppressor homolog 1/2 (Lats1/2) kinase-mediated phosphorylation of YAP1/TAZ triggers exclusion of the transcriptional coactivators from the nucleus and promotes their proteolytic degradation^20^. In the endothelium of most organs, YAP1/TAZ activity promotes angiogenic vessel growth, but, surprisingly, the two coactivators suppress EC proliferation and postnatal angiogenesis in bone^21^.

Here we demonstrate that EC-specific loss of the *Lats2* gene in ECs leads to extensive changes in the adult bone vasculature, EndMT and increased expression of ECM molecules and paracrine (also termed angiocrine) factors. These EC-specific changes promote the differentiation of metaphyseal BMSCs into myofibroblasts, resulting in bone fibrosis, osteosclerotic bone formation, impaired BM function and EMH. Loss of endothelial *Lats2* also impairs the formation of cartilage-resorbing septoclasts and results in the accumulation of chondrocytes (CHOs) in the mutant metaphysis. Mutant defects appear to be confined to the skeletal system, presumably due to compensation by LATS1 in other organs. These findings and the analysis of mouse models of myelofibrosis support that ECs play an instrumental role in bone fibrosis and need to be considered in disease etiology and in the context of therapeutic approaches.

## Results

### Endothelial LATS2 prevents bone vessel defects and fibrosis

**Fig. 1:**
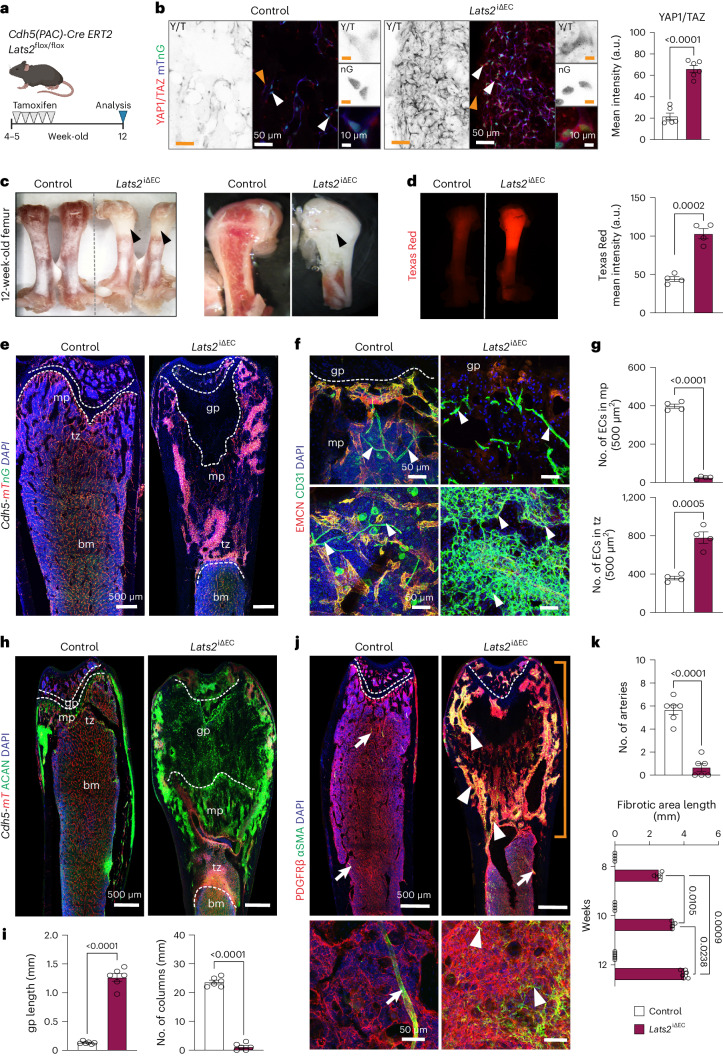
Endothelial Lats2 controls bone homeostasis and fibrosis. **a**, Scheme showing tamoxifen-induced *Lats2* inactivation in ECs with *Cdh5-CreERT2* transgenic mice. **b**, Representative confocal images showing increased YAP1/TAZ immunostaining in *Lats2*^iΔEC^ mutant bones. Endothelial YAP1/TAZ expression and nuclear localization were quantified (*n* = 6) by the mean intensity (a.u.) on the right side. Data are presented as mean ± s.e.m. *P* values, Mann–Whitney test (two-tailed). **c**, Freshly dissected 12-week-old control and *Lats2*^iΔEC^ mutant femurs (left) and longitudinal cross-section (right). Black arrowheads highlight pale areas. **d**, Microscopy images showing extravasation of Texas Red in control and *Lats2*^iΔEC^ mutant femur. On the right, the mean intensity (a.u.) of Texas Red is quantified (*n* = 4). Data are presented as mean ± s.e.m. *P* values, Mann–Whitney test (two-tailed). **e**, Confocal tile scan longitudinal view of *Lats2*^iΔEC^ and control femur in *Cdh5-mTnG* background. ECs, red (dTomato) and green (nuclear H2B-GFP), DAPI (blue). **f**,**g**, Maximum intensity projections of EMCN^+^ (red) and CD31^+^ (green) vessels in the femoral metaphysis (mp) (top) and transition zone (tz) (bottom). Note the abundance of mutant CD31^+^ EMCN^−^ ECs (**f**). Number of vessels and ECs is significantly reduced in the *Lats2*^iΔEC^ mp and increased in the tz area compared to control (**g**). Data (*n* = 4) are presented as mean ± s.e.m. *P* values, Mann–Whitney test (two-tailed). **h**,**i**, Tile scan confocal image showing avascular region filled with Aggrecan^+^ (ACAN; green) chondrocytes in *Lats2*^iΔEC^ metaphysis (**h**). Quantification showing increased growth plate (gp) length and loss of type H vessel columns in *Lats2*^iΔEC^ mp (*n* = 6). Presented as mean ± s.e.m. *P* values, Mann–Whitney test (two-tailed) (**i**). **j**, Tile scan confocal image and high-magnification image (below) showing widespread expression of PDGFRβ (red) and αSMA (green, arrowheads) in *Lats2*^iΔEC^ mutant femur. αSMA signal in control is confined to arterial SMCs. **k**, Quantification of arteries (*n* = 6) and length of fibrotic area (8-week-old and 10-week-old mice, *n* = 4; 12-week-old mice, *n* = 6). Mean ± s.e.m. *P* values, Mann–Whitney test (two-tailed) and Tukey multiple comparison test (one-way ANOVA). *n* indicates the number of independent biological samples. Source data

We increased YAP1/TAZ protein stability in ECs by deleting the upstream negative regulator LATS2 during bone remodeling. For this purpose, the tamoxifen-inducible, EC-specific *Cdh5-CreERT2* transgene was introduced into animals carrying a loxP-flanked (floxed) version of the *Lats2* gene. Tamoxifen administration for 5 d in a row at the age of 4–5 weeks generated *Lats2* loss-of-function mutants (*Lats2*^iΔEC^), which were analyzed at the age of 12 weeks (Fig. 1a). Survival, body weight and the external appearance of *Lats2*^iΔEC^ mutant mice is normal, and YAP1/TAZ immunostaining in the bone vasculature is increased relative to littermate controls (Fig. 1b and Extended Data Fig. 1a). Endothelial expression of *Lats2*, which is most prominent in arteries and metaphyseal (type H) vessels, is strongly reduced in *Lats2*^iΔEC^ mutants both at the transcript and protein level (Extended Data Fig. 1b,c). The metaphysis of freshly isolated *Lats2*^iΔEC^ femurs appears whiteish, indicating a decrease of red marrow, and shows enhanced vascular leakage (Fig. 1c,d). The *Lats2*^iΔEC^ bone vasculature is substantially altered. Metaphyseal capillaries are very sparse, and an abnormally dense network of CD31^+^ ECs is visible in the transition zone connecting the metaphysis to the adjacent diaphysis (Fig. 1e–g and Extended Data Fig. 1d,e). Capillaries of the mutant metaphysis and transition zone show elevated CD31 immunostaining but decreased EMCN signal, have a small diameter or lack a lumen and express CAV1 (Caveolin 1), a caveolar protein that is highly abundant in control arterial ECs (Fig. 1f and Extended Data Fig. 1e). The *Lats2*^iΔEC^ chondro-osseous junction next to the growth plate is strongly disorganized, and Aggrecan^+^ hypertrophic CHOs occupy a large region of the metaphysis, which is confirmed by both Safranin O histological staining and Collagen X immunostaining (Fig. 1h,i and Extended Data Fig. 2a,b).

CHO resorption is mediated by vessel-associated FABP5^+^ septoclasts, which show high expression of metalloproteinases, including MMP9 (refs. ^22,23^). FABP5^+^ cells and MMP9 immunosignals are concentrated at the control chondro-osseous junction but are lost in *Lats2*^iΔEC^ mutant samples (Extended Data Fig. 2c). Another striking alteration in the *Lats2*^iΔEC^ mutant metaphysis is the strong upregulation of markers associated with tissue scarring and fibrosis, such as platelet-derived growth factor receptor β (PDGFRβ) and α-smooth muscle actin (αSMA^+^) (Fig. 1j,k and Extended Data Fig. 2d,e). Furthermore, AZAN trichrome and Reticulin histological staining, together with immunostaining for the ECM protein Collagen fibers, indicate widespread fibrosis in the *Lats2*^iΔEC^ metaphysis (Extended Data Fig. 2f,g).

To gain insight into the emergence of vascular defects and fibrosis in *Lats2*^iΔEC^ mutant bone, we analyzed samples at 8 weeks and 10 weeks of age. Increased perivascular αSMA expression is already visible at 8 weeks, and the disorganized area is further increased by 10 weeks (Extended Data Fig. 3a,b). Changes in the vasculature of the metaphysis and the transition zone bordering the diaphysis follow a similar timecourse (Extended Data Fig. 3c,d). Remarkably, fibrosis and vascular alterations in 12-week-old mutants are confined to bone, whereas no overt defects can be seen in kidney, lung, liver and heart sections (Extended Data Fig. 3e). Thus, loss of endothelial LATS2 leads to organ-specific defects in bone.

### Osteosclerotic defects after loss of endothelial LATS2

**Fig. 2:**
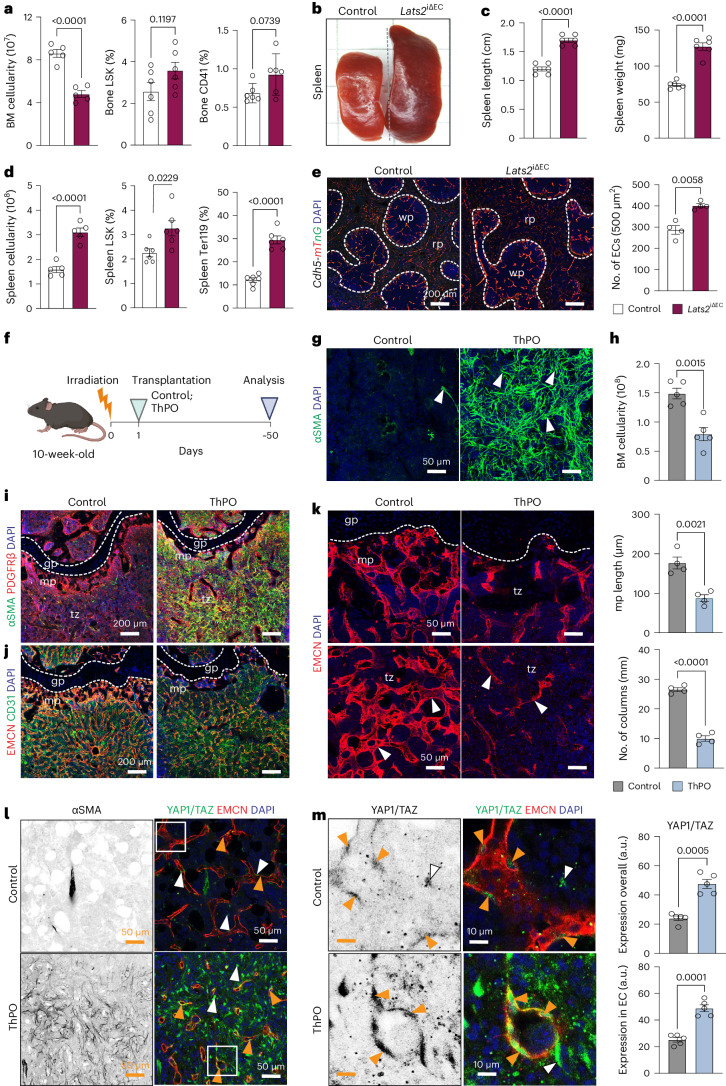
Loss of LATS2 promotes EMH. **a**, Quantification of BM cellularity and FACS analysis of LSK and CD41^+^ cell frequency (*n* = 6). Mean ± s.e.m. *P* values, Mann–Whitney test (two-tailed). **b**,**c**, Images showing freshly dissected *Lats2*^iΔEC^ and control spleen (**b**) and quantification of spleen size and weight (**c**) (*n* = 6). Data are presented as mean ± s.e.m. *P* values, Mann–Whitney test (two-tailed). **d**, Quantification of spleen cellularity and frequency of LSK and Ter119 cells (*n* = 6). Data are presented as mean ± s.e.m. *P* values, Mann–Whitney test (two-tailed). **e**, Sections of control and *Lats2*^iΔEC^ spleens in *Cdh5-mTnG* reporter background. Red pulp (rp) and white pulp (wp) are indicated. Nuclei, DAPI (blue). Quantification showing increased EC number in *Lats2*^iΔEC^ mutant (*n* = 4). Data are shown as mean ± s.e.m. *P* values, Mann–Whitney test (two-tailed). **f**, Scheme showing ThPO-induced BM fibrosis. **g**, Representative confocal images showing αSMA (green) immunostaining in ThPO and control bone. Nuclei, DAPI (blue). **h**, Quantification of BM cellularity of control and ThPO mice (*n* = 5). Data are presented as mean ± s.e.m. *P* values, Mann–Whitney test (two-tailed). **i**,**j**, Representative confocal images showing increase of metaphyseal αSMA signal (**i**) and reduction of EMCN^+^ CD31^+^ vessels (**j**) in ThPO femur relative to control. **k**, EMCN^+^ ECs (red) in metaphysis (mp) (top) and diaphysis (dp) (bottom). Nuclei, DAPI (blue). Graphs on the right show decreased mp length and vessel columns after ThPO (*n* = 4). Data are shown as mean ± s.e.m. *P* values, Mann–Whitney test (two-tailed). **l**,**m**, Representative single optical confocal images showing increased expression of αSMA (gray) and YAP1/TAZ (green) in EMCN (red) ECs (orange arrowheads) in ThPO bone (**l**). Cropped zoomed-in image displays YAP1/TAZ nuclear (DAPI, blue) localization in ECs (**m**). Quantification shows increase of overall and EC-specific Yap1/TAZ immunosignal in ThPO bones (*n* = 5). Data are shown as mean ± s.e.m. *P* values, Mann–Whitney test (two-tailed). *n* indicates the number of independent biological samples. Source data

Bone fibrosis is known to cause the displacement of hematopoietic cells from BM, which leads to progressive splenomegaly due to EMH^24^. Analysis of *Lats2*^iΔEC^ peripheral blood shows an elevation of white blood cells, whereas red blood cells and platelets are similar to control (Extended Data Fig. 4a). In *Lats2*^iΔEC^ BM, total cellularity and Ter119^+^ erythrocytes and B220^+^ B lymphocytes are decreased, whereas Lin^−^Sca-1^+^c-Kit^+^ (LSK) hematopoietic stem and progenitor cells (HSPCs) are not significantly altered (Fig. 2a and Extended Data Fig. 4b). CD41^+^ megakaryocytes are known to act as main drivers of myelofibrosis^24^, but these cells are absent from the *Lats2*^iΔEC^ fibrotic area, and their total number is not significantly altered in mutant femurs due to their presence in the diaphyseal BM (Fig. 2a and Extended Data Fig. 4c,d). Consistent with EMH, *Lats2*^iΔEC^ mutants show increased spleen volume and cellularity together with an increase in splenic HSPCs and Ter119^+^ cells, whereas the fractions of CD45^+^ leukocytes and B220^+^ cells are reduced (Fig. 2b–d and Extended Data Fig. 4e). In spleen sections, the area of red pulp is increased relative to white pulp, a feature of EMH (Fig. 2e and Extended Data Fig. 4f,g). These alterations are likely to be secondary to the defects in *Lats2*^iΔEC^ BM, as YAP1/TAZ immunostaining in splenic vessels is not increased (Extended Data Fig. 4h). Thus, *Lats2*^iΔEC^ mutants display splenomegaly, BM fibrosis and impaired BM function, resembling key features of myelofibrosis.

The etiology of myelofibrosis in human subjects is poorly understood, but previous work established that BM fibrosis in mice can be induced by overexpressing ThPO from hematopoietic cells, which results in the expansion of megakaryocytes, elevated expression of platelet-derived growth factor and transforming growth factor β and the activation of mesenchymal stromal cells^10,25^. ThPO overexpression in HSPCs first leads to leukocytosis and thrombocytosis and, over time, with onset of fibrosis, reduced hemoglobin and erythrocyte counts (Fig. 2f and Extended Data Fig. 5a). The ThPO overexpression model mirrors aspects of the *Lats2*^iΔEC^ phenotype: increased and widespread αSMA immunostaining reflecting fibrosis, a general loss of cellularity in bone as well as an increase in spleen weight (Fig. 2g–i and Extended Data Fig. 5b,d). Remarkably, ThPO overexpression also induces a marked reduction of metaphyseal type H vessels and lowers endothelial EMCN expression (Fig. 2j,k and Extended Data Fig. 5c). Consistent with the loss of type H vessels, perivascular OSX^+^ OB lineage cells are strongly reduced in long bones of ThPO-overexpressing mice (Extended Data Fig. 5e). Strikingly, ThPO induces a strong increase in YAP1/TAZ immunostaining in fibrotic BM (Extended Data Fig. 5f), but it also significantly enhances YAP1/TAZ in ECs relative to control (Fig. 2l,m). Somatic activating mutations (such as W515L) in the transmembrane domain of the ThPO receptor, encoded by the *MPL* gene, are a driver of primary myelofibrosis in humans. Transplantation of MPL^W515L^-expressing hematopoietic cells can reproduce major features of the disease, including splenomegaly and extramedullary hematopoiesis, in mice^26^. In the MPL^W515L^ model, fibrosis is accompanied by widespread upregulation of αSMA (Extended Data Fig. 5g,h). Strikingly, the EMCN^+^ BM vasculature of these mice is significantly reduced, and YAP1/TAZ immunostaining in vessels is upregulated (Extended Data Fig. 5h,i). These data show that vascular alterations and upregulation of YAP1/TAZ are a common feature in two established models of myelofibrosis.

**Fig. 3:**
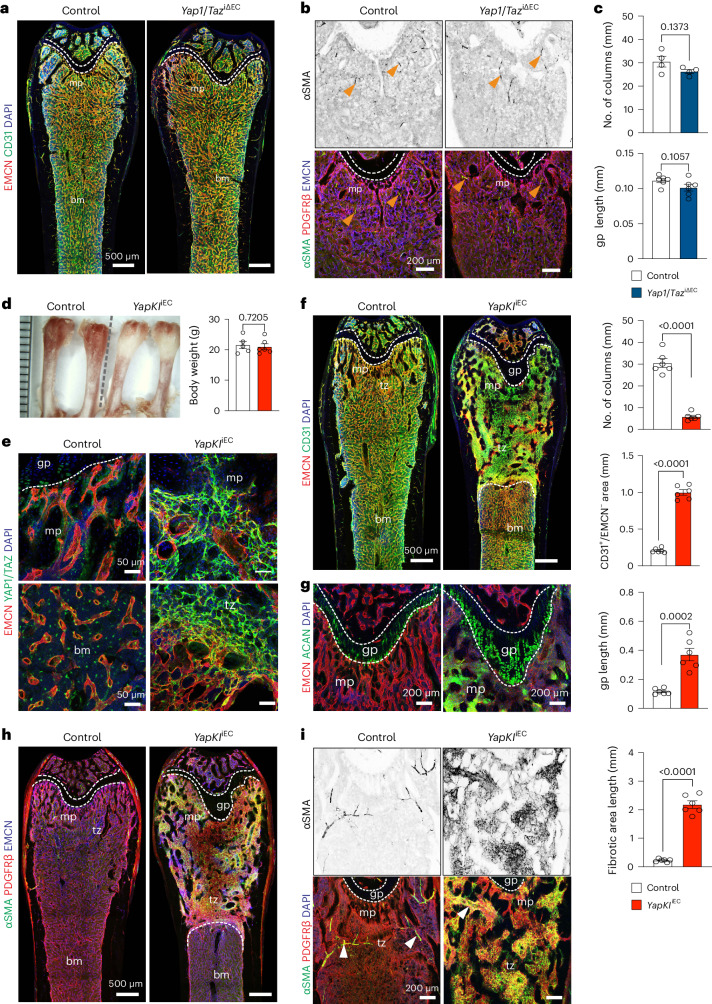
Overexpression of a stabilized Yap1 in ECs promotes BM fibrosis. **a**, Tile scan confocal overview images of EMCN (red) and CD31 (green) immunostained vasculature in control and *Yap1/Taz*^iΔEC^ compound mutant femur. Metaphysis (mp), bone marrow (bm) and growth plate (gp) (dashed lines) are indicated. **b**,**c**, Representative confocal image of femoral αSMA^+^ (green) smooth muscle cells covering arteries (arrows) (above). EMCN^+^ (blue) blood vessels and PDGFRβ^+^ cells (red) in *Yap1/Taz*^iΔEC^ mutant and littermate control (below). Metaphyseal vessel columns and gp are not altered in *Yap1/Taz*^iΔEC^ bone (**c**) (*n* = 4, 6). Data are shown as mean ± s.e.m. *P* values, Mann–Whitney test (two-tailed). **d**, Freshly dissected 12-week-old *YapKI*^iEC^ gain-of-function and control femurs. Quantitative analysis of *YapKI*^iEC^ and littermate control body weight (*n* = 5). Data are shown as mean ± s.e.m. *P* values, Mann–Whitney test (two-tailed). **e**, Representative confocal images showing increased YAP1/TAZ (green) expression in *YapKI*^iEC^ blood vessels (EMCN^+^, red) relative to control. **f**. Tile scan longitudinal view of EMCN and CD31 immunostaining in femur sections. Quantification shows decrease in number of vessel columns and increase in CD31^+^/EMCN^−^ vascular area in *YapKI*^iEC^ mutant (*n* = 6). Data are shown as mean ± s.e.m. *P* values, Mann–Whitney test (two-tailed). **g**, Representative confocal images showing increase in Aggrecan^+^ (ACAN^+^) chondrocytes in *YapKI*^iEC^ femur. Graph shows increased size of gp relative to control (*n* = 6). Data are shown as mean ± s.e.m. *P* values, Mann–Whitney test (two-tailed). **h**,**i**. Tile scan confocal longitudinal view of PDGFRβ^+^ (red) and αSMA^+^ (green) cells. αSMA in control sections is largely confined to arterial smooth muscle cells (**h**). High-magnification images of αSMA^+^ (gray) fibrotic area is shown on the top right. gp (dashed line), metaphysis (mp) and transition zone (tz) to BM are indicated (**i**) (*n* = 6). Data are shown as mean ± s.e.m. *P* values, Mann–Whitney test (two-tailed). *n* indicates the number of independent biological samples. Source data

LATS2 and the related LATS1 kinase phosphorylate YAP1 and TAZ, which limits the activity of the two transcriptional coregulators due to cytoplasmic retention and proteasomal degradation. To directly address the role of YAP1 and TAZ, we generated tamoxifen-inducible, EC-specific loss-of-function double mutant mice (*Yap1/Taz*^iΔEC^), which were given tamoxifen at the age of 4–5 weeks before analysis at 12 weeks. In contrast to the enhanced developmental angiogenesis in postnatal *Yap1/Taz*^iΔEC^ bone^21^, adult mutants display a normal organization of the bone vasculature and also lack alterations in PDGFRβ and αSMA expression (Fig. 3a–c). Overexpression of stabilized YAP1 (YAP1^S112A^) in ECs, however, causes the formation of sclerotic lesions, vascular impairment and the appearance of an EMCN-deficient endothelial network in the metaphysis of the resulting *YapKI*^iEC^ gain-of-function mutants (Fig. 3d–f). Key defects observed in *Lats2*^iΔEC^ mutants, such as expansion of chondrocytes and widespread expression of PDGFRβ and αSMA, are also observed in *YapKI*^iEC^ animals (Fig. 3g–i).

**Fig. 4:**
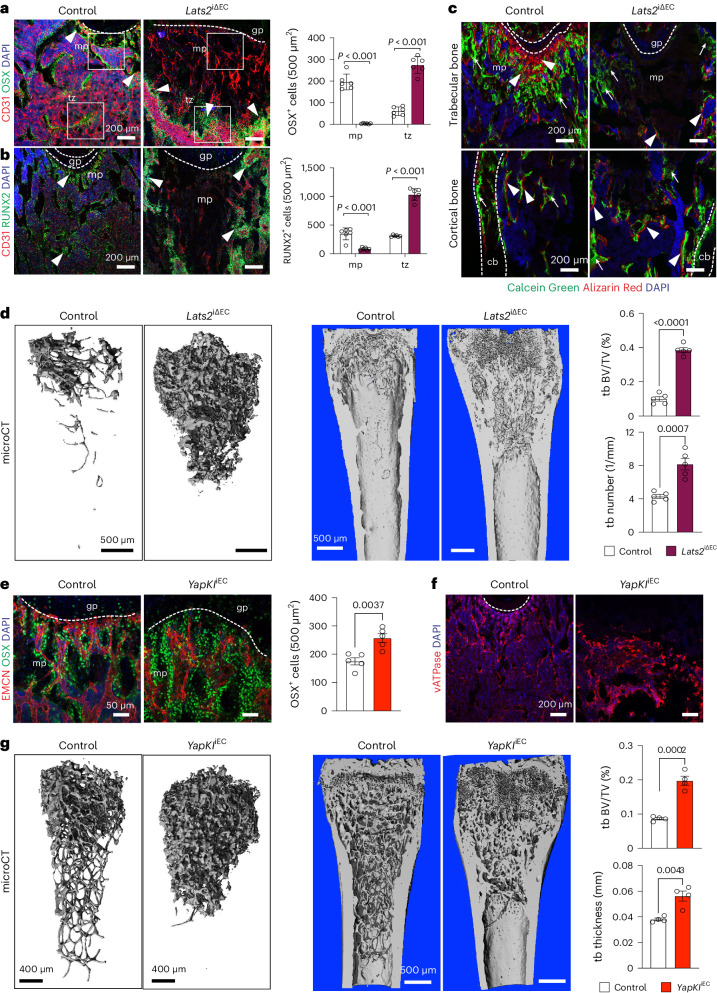
Endothelial Hippo signaling controls osteosclerosis. **a**,**b**, Representative confocal images showing OSX^+^ cells (**a**) and RUNX2^+^ osteoprogenitors (**b**) (arrowheads) in 12-week-old *Lats2*^iΔEC^ femur and littermate control (*n* = 6). Data are shown as mean ± s.e.m. *P* values, Mann–Whitney test (two-tailed). **c**, Representative confocal image showing double labeling with Calcein Green and Alizarin Red in metaphysis (mp) (top) and border to diaphysis (dp) (bottom) in control and *Lats2*^iΔEC^ femur. Arrow highlights Calcein Green deposition, and arrowheads show double labeling of deposition/resorption. **d**, Representative µCT images of *Lats2*^iΔEC^ and control femoral trabecular bone (tb). Quantitative analysis of tb volume (bone volume/total volume (BV/TV)) and number are shown on the right (*n* = 5). Data are shown as mean ± s.e.m. *P* values, Mann–Whitney test (two-tailed). **e**,**f**, Representative confocal image showing OSX^+^ cells in control and *YapKI*^iEC^ femoral metaphysis (*n* = 5). Data are shown as mean ± s.e.m. *P* values, Mann–Whitney test (two-tailed) (**e**). vATPase^+^ osteoclasts (red) are reduced in the *YapKI*^iEC^ fibrotic area (**f**). **g**, Representative µCT images of tb in control and *YapKI*^iΔEC^ femur. Quantitative analysis of tb volume (BV/TV) and number (*n* = 4). Data are presented as mean ± s.e.m. *P* values, Mann–Whitney test (two-tailed). *n* indicates the number of independent biological samples. Source data

Previous work showed that metaphyseal type H capillaries are associated with osteoprogenitor cells and actively promote osteogenesis^16^. OSX^+^ cells and RUNX2^+^ osteoprogenitors are strongly reduced in the *Lats2*^iΔEC^ metaphysis in regions that have lost capillaries. In contrast, OB lineage cells are enriched around the dense but disorganized endothelial network in the mutant transition zone (Fig. 4a,b). Analysis of bone remodeling using the Calcein Green and Alzarin Red double labeling approach (Methods) shows decreased osteogenesis and resorption in *Lats2*^iΔEC^ long bone (Fig. 4c). Vacuolar ATPase (vATPase)-expressing osteoclasts are decreased in the avascular metaphysis and elevated in the mutant transition zone (Extended Data Fig. 6a). Microcomputed tomography (µCT) shows highly increased trabecular bone volume, quantity and thickness in *Lats2*^iΔEC^ mutant femurs, whereas cortical bone thickness is not changed (Fig. 4d and Extended Data Fig. 6b,c). Supporting that the role of LATS2 in bone endothelium involves the modulation of YAP1/TAZ, *YapKI*^iEC^ gain-of-function increases the number of osteoprogenitors, alters the distribution of osteoclasts and leads to sclerotic mineralization (Fig. 4e–g). In contrast, adult *Yap1/Taz*^iΔEC^ loss-of-function mutants show a modest decrease of OSX^+^ osteoprogenitors, whereas bone mineralization is unaffected (Extended Data Fig. 6d,e). These findings indicate that YAP1/TAZ levels are normally kept low in the adolescent/adult bone endothelium so that EC-specific overexpression of YAP1 or loss of LATS2 severely impair bone remodeling and homeostasis.

### LATS2 deletion induces EndMT

**Fig. 5:**
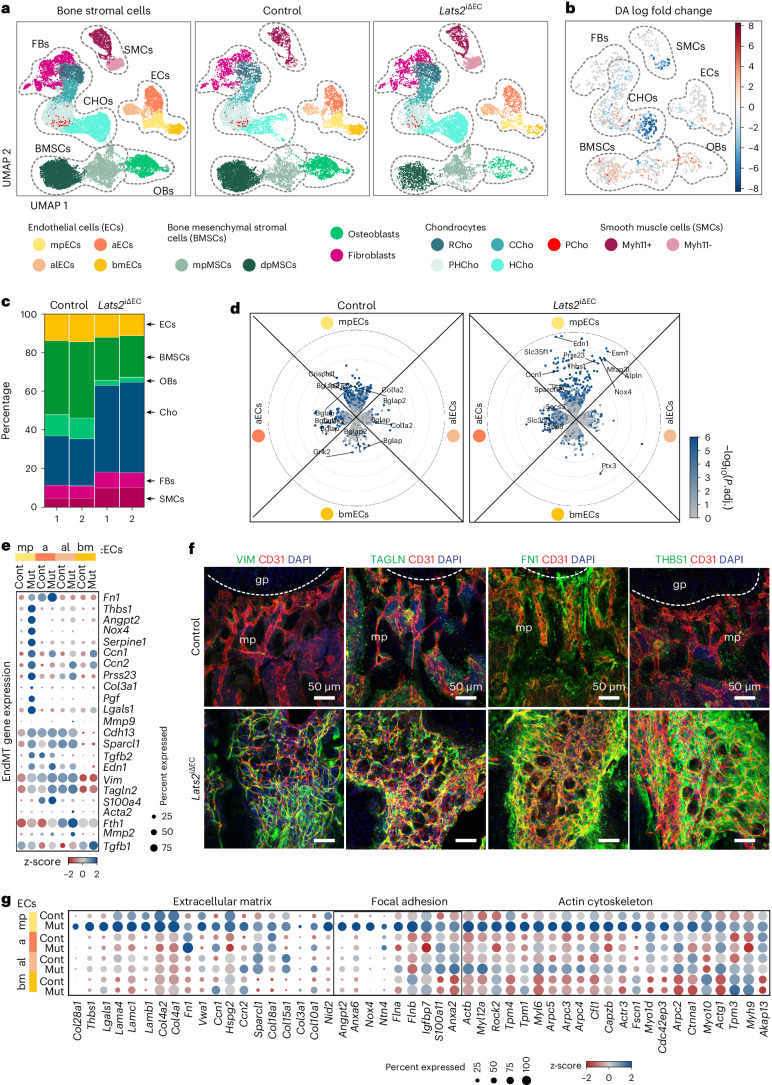
EndMT after loss of LATS2. **a**,**b**, UMAP plots showing merged (left) and separated control and *Lats2*^iΔEC^ mutant (right) bone stromal cells color-coded by cell type (**a**). Changes in cellular abundance (differential abundance (DA)) as calculated with milopy. Negative values indicate increased abundance in *Lats2*^*iΔEC*^ mutant (**b**). Metaphyseal ECs (mpECs), arteriole-like ECs (alECs), arterial ECs (aECs), metaphyseal MSCs (mpMSCs, Osteo-CAR), diaphyseal MSCs (dpMSCs, Adipo-CAR), resting chondrocytes (RCHOs), columnar chondrocytes (CCHOs), proliferating chondrocytes (PCHOs), pre-hypertrophic chondrocytes (PHCHOs), hypertrophic chondrocytes (HCHOs) as well as Myh11^+^ and Myh11^−^ SMCs are indicated. **c**, Bar graph displaying percentage of each main bone stromal cell type. **d**, Radial expression plot (Methods) showing DGE of control and *Lats2*^iΔEC^ EC subclusters. *y* axis shows log_2_ fold expression changes; color scale represents the adjusted *P* values. **e**, Dot plot for normalized and averaged expression indicates high expression of mesenchymal markers and fibroblast genes in *Lats2*^iΔEC^ mpECs. z-scaled per row. **f**, Immunostaining showing high levels of Vimentin (VIM), Transgelin (TAGLN), Fibronectin 1 (FN1) and Thrombospondin 1 (THBS1) in *Lats2*^iΔEC^ metaphysis (mp). Control growth plate (gp) is marked by the dashed line. ECs (CD31, red) and nuclei (DAPI, blue) are labeled. **g**, Row-wise z-scaled normalized expression values of ECM, focal adhesion and actin cytoskeleton genes in *Lats2*^iΔEC^ mutant and control EC populations. FB, fibroblast.

To determine stromal cell heterogeneity, fate specification and cellular crosstalk in *Lats2*^iΔEC^ mutant and littermate control long bone, we performed droplet-based single-cell RNA sequencing (scRNA-seq) of non-hematopoietic bone stromal cells with the 10x Chromium platform. We obtained a total of 20,184 cells after removing doublets and residual hematopoietic cells from scRNA-seq experiments of control and *Lats2*^iΔEC^ mutants with two biological replicates each. Based on the expression of known marker genes, we identified six primary cell types: ECs, BMSCs, OBs, smooth muscle cells (SMCs), CHOs and fibroblasts as well as their subpopulations. BMSC numbers are decreased and CHO and SMC numbers are increased in *Lats2*^iΔEC^ mutants (Fig. 5a–c and Extended Data Fig. 7a–c). Within the EC cluster, metaphyseal (type H) ECs (mpECs, Ramp3^+^), bone marrow (type L) ECs (bmECs, Stab2^+^), arteriole-like ECs (alECs, Fmo2^+^) and arterial ECs (aECs, Gja4^+^) can be readily identified (Extended Data Fig. 7d–f). When compared to controls, *Lats2*^iΔEC^ ECs exhibit numerous downregulated but also strongly upregulated differentially expressed genes (DEGs) (Extended Data Fig. 7g). *Lats2*^iΔEC^ mutant mpEC and aEC subpopulations show particularly large numbers of upregulated genes (Fig. 5d). Expression of the YAP1/TAZ-controlled target genes *Ccn1* (encoding cysteine-rich angiogenic inducer 61 (CYR61)), *Ccn2* (connective tissue growth factor (CTGF)), *Angpt1* (angiopoietin 1) and *Thbs1* (thrombospondin 1) is strongly increased (Extended Data Fig. 7h). Surprisingly, genes typical for mesenchymal cells and fibroblasts are also strongly upregulated in *Lats2*^iΔEC^ mpECs. The differential expression of a subset of these genes, including *Vim* (encoding the intermediate filament protein vimentin), *Tagln* (smooth muscle 22α, SM22α), *Fn1* (fibronectin 1), *Thbs1* and *Angpt2* is confirmed by immunostaining of the corresponding gene products in bone sections (Fig. 5e,f and Extended Data Fig. 8a). Additionally, we noticed that *Lats2*^iΔEC^ mutant type H ECs (mpECs) show expression of gene signatures associated with ECM, cell–matrix adhesion and actin cytoskeleton (Fig. 5g and Extended Data Fig. 8b). Furthermore, immunostaining of two critical downstream regulators controlling cell–matrix interactions, active integrin β1 and phosphorylated focal adhesion kinase (FAK), is strongly increased (Extended Data Fig. 8b). Ultrastructural analysis by transmission electron microscopy confirms the profoundly increased deposition of matrix components in the *Lats2*^iΔEC^ metaphysis and pronounced actin fibers in mutant ECs (Extended Data Fig. 8c). Genetic lineage tracing supports that *Lats2*^iΔEC^ ECs are not fully converted into mesenchymal stromal cells (Extended Data Fig. 8d) but acquire a mesenchymal cell-like phenotype through EndMT, including the expression of typical mesenchymal markers and elevated cell–matrix interactions.

### Endothelial signals control myofibroblast generation

**Fig. 6:**
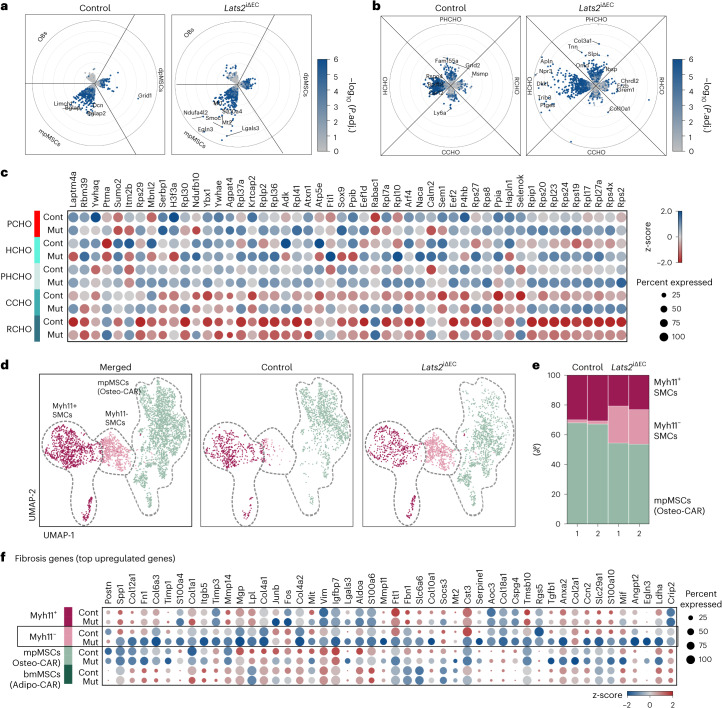
EC-derived signals control mpMSCs and CHOs. **a**–**c**, Radial expression plot (Methods) of BMSC/OB subsets, showing that most significant differences in regulation between conditions occur in mpMSCs (**a**). Within the CHO subset, most differences between conditions are confined to HCHOs. Proliferating CHOs are not shown, as number was less than 50 (**b**). Genes significantly differentially expressed (*P*.adj ≤ 0.01, log_2_FC < 0, frac. expressed ≥ 0.2) between control (Cont) and mutant (Mut) in at least one CHO sub-identity (**c**). **d**,**e**, UMAP visualization of mpMSC and SMC subclusters showing all cells (left) or split by genotype (right) (**d**). Percentage of cells in each cell type per sample is shown. Note increase in *Lats2*^iΔEC^ Myh11^−^ SMCs representing myofibroblasts (**e**). **f**, Row-wise z-scaled normalized expression values of fibrosis genes in Myh11^−^ cells relative to mpMSCs, dpMSCs and Myh11^+^ SMCs.

**Fig. 7:**
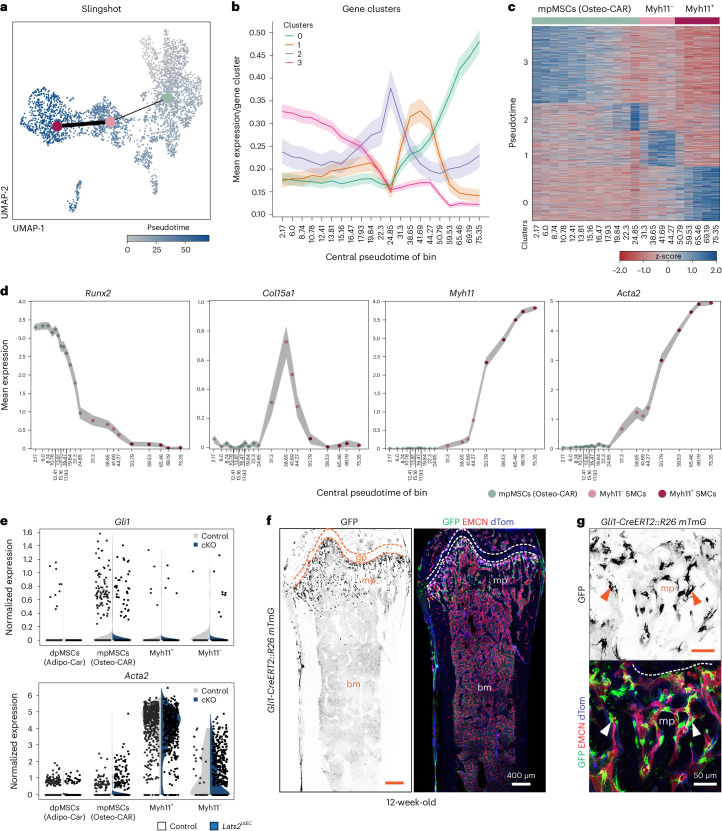
Conversion of mpMSCs into myofibroblasts. **a**–**c**, UMAP plot colored by slingshot pseudotime. Centroid dots show cell type identity, and connecting lines indicate cell type connectivities calculated by PAGA (**a**). Visualization of mean expression in gene clusters as determined by Leiden clustering genes along the slingshot pseudotime trajectory; filled areas show 95% confidence interval (**b**). Heatmap of these gene clusters showing gene expression changes across pseudotime (**c**). **d**, Selected marker genes showing continuous gene expression changes across the pseudotime trajectory from mpMSC to Myh11^+^ SMC. Normalized expression counts averaged per pseudotime bin are shown; filled areas show 95% confidence interval. **e**, Violin plots showing *Gli1* and *Acta2* expression in dpMSCs/Adipo-CAR cells, mpMSCs/Osteo-CAR cells as well as Myh11^+^ and Myh11^−^ SMCs of *Lats2*^iΔEC^ mutants relative to control. **f**,**g**, Longitudinal tile scan confocal images showing *Gli1-CreERT2*-labeled cells (green) in the metaphysis (**f**). High-magnification images confirm perivascular location of GFP^+^ cells (**g**). EMCN (red) and dTomato (blue).

Previous research revealed that *Lepr-Cre*^+^ and *Gli1*^+^ mesenchymal stromal cells give rise to myofibroblasts during hematopoietic cell-induced myelofibrosis^9,10^. As the exact nature of the processes leading to bone fibrosis is incompletely understood, we analyzed gene expression changes in different cell populations. We noted that transcriptional alterations in *Lats2*^iΔEC^ mpMSCs (Osteo-CAR) cells are more extensive than those seen in diaphyseal mesenchymal stromal cells (dpMSCs; Adipo-CAR cells) or OBs (Fig. 6a). In the CHO cluster, pre-hypertrophic chondrocytes (PHCHOs) and hypertrophic chondrocytes (HCHOs) show the largest number of DEGs (Fig. 6b,c). We also noted a strong enrichment of *Myh11*^−^ cells in the *Lats2*^iΔEC^ SMC cluster, which show increased expression of markers associated with fibrosis and are likely to represent myofibroblasts (Fig. 6d–f). Analysis of the same scRNA-seq data also shows a pronounced shift within the *Lats2*^iΔEC^ mpMSC subcluster, indicative of altered gene expression relative to control (Fig. 6d). Pseudotime^27^ and partition-based graph abstraction (PAGA)^28^ analysis of the *Lats2*^iΔEC^ scRNA-seq data suggest a potential differentiation trajectory from mpMSCs to *Myh11*^−^ cells in the SMC cluster (Fig. 7a–d). Although *Gli1* expression is most prominent in the mpMSC/Osteo-CAR subcluster, *Acta2* transcripts (encoding αSMA) are abundant in *Myh11*^+^ SMCs and strongly upregulated in *Lats2*^iΔEC^ *Myh11*^*−*^ SMCs/myofibroblasts (Fig. 7e). Consistent with previous studies highlighting the role of Gli1^+^ cells as a source of myofibroblasts in bone fibrosis^10^, *Gli1-CreERT2*-mediated recombination of a Cre reporter allele labels vessel-associated BMSCs in the 12-week-old metaphysis, namely mpMSC/Osteo-CAR cells (Fig. 7f,g).

**Fig. 8:**
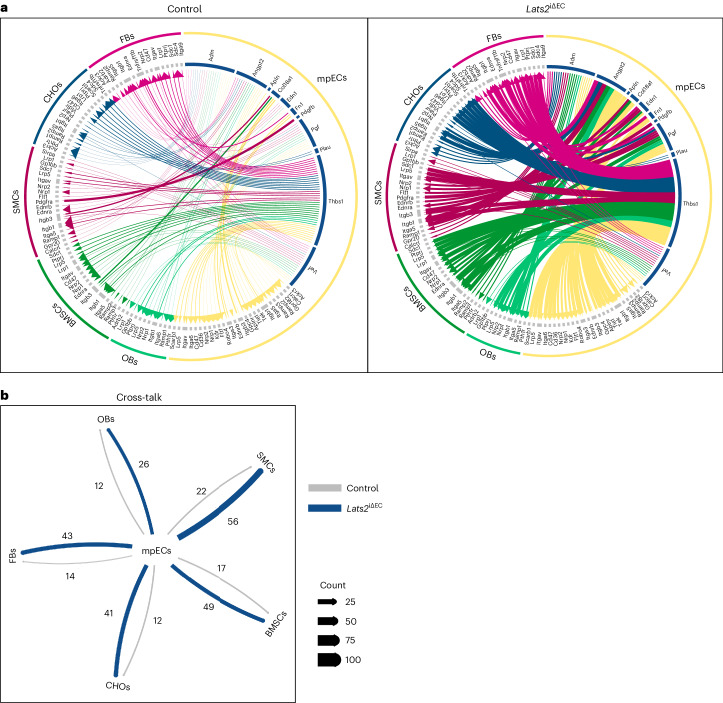
Loss of Lats2 increases endothelial signaling interactions. **a**, Ligand-receptor pairings indicating increased mpECs signaling to stromal cells in *Lats2*^iΔEC^ mutants. Relative (min-max normalized) changes in normalized expression counts of ligand and receptor for selected interactions (LRI) (scaled between 0 and 1) originating from mpECs. Interactions are significant at *P*.adj of LRI score in at least one condition and LRIDiffScore ≤ 0.05 and LRIDiff ≥ 1. **b**, Number of significant LRIs between cell types of interest (*P*.adj. LRIScore ≤ 0.05; *P*.adj. LRIDiffScore ≤ 0.05; |LRIDiffScore| ≥ 1). FB, fibroblast.

Next, we sought to understand which EC-derived signals might lead to the conversion of mpMSC/Osteo-CAR cells into myofibroblasts and the emergence of fibrosis in the *Lats2*^iΔEC^ metaphysis. To identify potential ligand–receptor interactions (LRIs), we employed a newly developed approach (Methods) similar to CellPhoneDB^29^. *Lats2*^iΔEC^ mutant mpECs show substantially increased paracrine signaling capability compared to other EC subtypes, reflected by strongly elevated expression of secreted molecules relative to control (Extended Data Fig. 9a). In turn, many of the respective receptors for these EC-derived ligands are expressed or even elevated in other cell types in *Lats2*^iΔEC^ bone (Fig. 8a,b and Extended Data Fig. 9b). Specifically, genes involved in cell–matrix interactions (*Thbs1*, *Fn1*, *Col4a1*, *Col18a1* and *Plau*), the glycoprotein von Willebrand factor (*Vwf*) and secreted signaling molecules, such as adrenomedullin (*Adm*), apelin (*Apn*), angiopoietin 2 (*Angpt2*) and endothelin 1 (*Edn1*), are upregulated in *Lats2*^iΔEC^ mutant mpECs (Extended Data Fig. 9c). The corresponding receptors are expressed by multiple cell populations but are frequently found in mpMSCs and the *Myh11*^−^ SMC subcluster (Extended Data Fig. 9c,d). Treatment of cultured BMSCs with recombinant endothelin 1 can increase the expression of fibrosis markers similar to TGFβ1, whereas adrenomedullin has no significant effect under the same conditions (Extended Data Fig. 9e). Based on these results, we propose that loss of LATS2 in ECs is sufficient to initiate bone fibrosis through the upregulation of EC-derived secreted molecules.

### YAP1/TAZ control EC plasticity through serum response factor

**Fig. 9:**
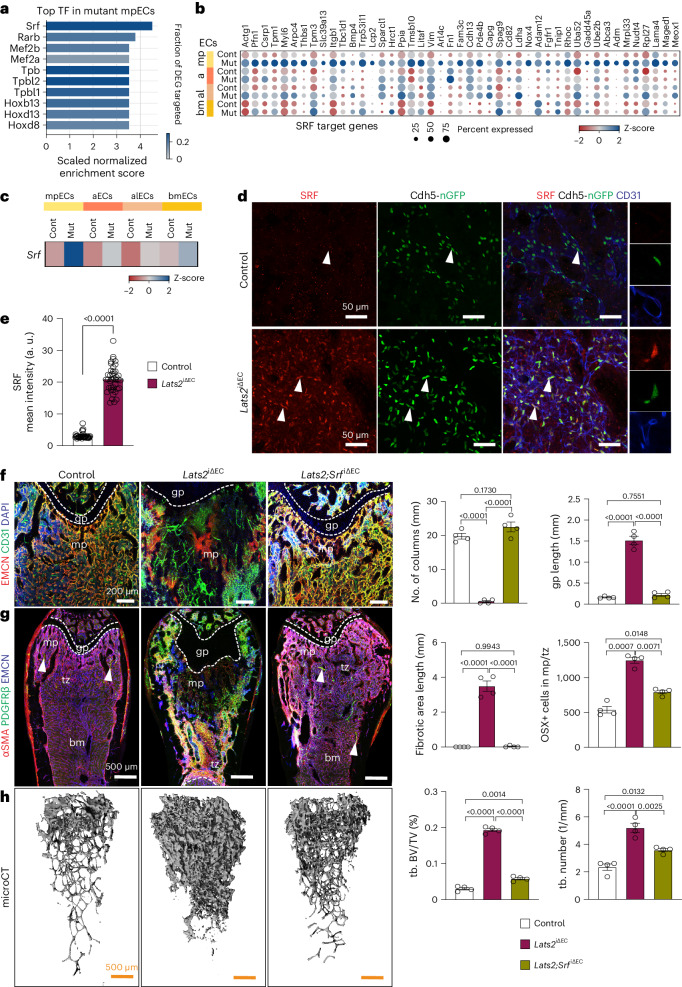
YAP1/TAZ regulate EC plasticity through SRF. **a**,**b**, Top transcription factors (TFs), predicted by iRegulon using genes significantly upregulated in *Lats2*^iΔEC^ mpECs (325 total genes; log_2_FC ≥ 1, *P*.adj ≤ 0.05, frac. expressed ≥ 0.2) compared to control (**a**). Row-wise z-scaled normalized expression values of known SRF target genes (**b**). **c**–**e**, *Srf* transcript levels in row-wise z-scaled normalized expression counts (**c**) and immunostaining signals (**d**) are strongly increased in *Lats2*^iΔEC^ mutant mpECs. Bar graph shows SRF mean intensity (a.u.) in ECs (**e**). Data (*n* = 4; 40 cells) are presented as mean ± s.e.m. *P* values, Mann–Whitney test (two-tailed). **f**, Maximum intensity projections of EMCN (red) and CD31 (green) immunostaining in control, *Lats2*^iΔEC^ and *Lats2 Srf* ^iΔEC^ double mutant femur. Quantification shows number of type H vessel columns and growth plate (gp) length. **g**, Tile scan confocal longitudinal view showing PDGFRβ and αSMA immunostaining. Arrowheads indicate arterial αSMA^+^ SMCs. Graphs show length of fibrotic area and number of OSX^+^ cells. **h**, Representative µCT images of femoral trabecular bone (tb). Quantitative analysis of tb volume (bone volume/total volume (BV/TV)) and number. Data for **f**–**h** (*n* = 4 each) are shown as mean ± s.e.m. *P* values, Tukey multiple comparison test (one-way ANOVA). *n* indicates the number of independent biological samples. Source data

To gain insight into the mechanism controlling EndMT in *Lats2*^iΔEC^ mutants, we used iRegulon^30^ to examine which transcription factors are likely regulators of the gene expression changes in mpECs. Serum response factor (SRF) is the top hit in this analysis, and SRF target genes are upregulated in *Lats2*^iΔEC^ mpECs (Fig. 9a,b). Further arguing for a potential role of SRF, transcript and protein are highly increased in mutant mpECs relative to control (Fig. 9c–e). *Srf* inactivation in ECs (*Srf* ^iΔEC^) alone has no significant effect on the bone vasculature, tissue organization, markers of fibrosis, growth plate size or mineralized bone, but CD31^+^/EMCN^−^ vessels, representing arteries/arterioles, are slightly reduced, and OSX^+^ cells are increased (Extended Data Fig. 10a–e). In EC-specific *Lats2* and *Srf* compound mutants (*Lats2 Srf* ^iΔEC^) generated with the same tamoxifen administration regime described earlier, the lack of SRF largely restores the normal organization of metaphyseal vasculature and EMCN expression in the *Lats2*^iΔEC^ background (Fig. 9f and Extended Data Fig. 10f–h). Vascular normalization is accompanied by the restoration of normal growth plate size and bone mineralization as well as reduction of PDGFRβ and αSMA immunostaining in *Lats2 Srf* ^iΔEC^ compound mutants (Fig. 9g,h and Extended Data Fig. 10i,j). The sum of these data shows that LATS2 limits YAP1/TAZ expression in bone ECs to prevent EndMT and SRF-mediated processes, leading to fibrosis and osteosclerotic defects (Extended Data Fig. 10k).

## Discussion

Our results demonstrate that loss of the LATS2 kinase endows bone ECs with disease-promoting properties, resulting in osteosclerotic defects, bone fibrosis, EMH and splenomegaly. This involves the upregulation of YAP1/TAZ, and, accordingly, EC-specific overexpression of stabilized YAP1^S112A^ protein phenocopies major aspects of the defects seen in *Lats2*^iΔEC^ loss-of-function bone. YAP1 and TAZ are well known for their prominent role in the regulation of cell proliferation and tissue growth. In the endothelium of most organs, combined inactivation of *Yap1* and *Taz* impairs angiogenic blood vessel growth^31,32^, whereas the opposite phenotype, namely vascular overgrowth, occurs in the postnatal bone vasculature^21^. Our findings presented here indicate that YAP1 and TAZ are no longer required in the adult bone vasculature, and, instead, it is critical for healthy homeostasis that the activity of the two transcriptional coregulators is actively suppressed by LATS2.

Remarkably, loss of LATS2 is not sufficient to cause defects in the endothelium of multiple other organs, which might reflect redundant activity of the related kinase LATS1. It has been shown that LATS1 and LATS2 act non-redundantly in some biological contexts but can often compensate for each other^33,34^. In ECs of the growing retina and brain, combined inactivation of *Lats1* and *Lats2* results in hyperproliferation and metabolic activation through upregulation of the proto-oncogene c-Myc, which is rescued by the simultaneous inactivation of *Yap1*/*Taz*^31^. In the bone endothelium, however, LATS1 is unlikely to be a major player because it cannot prevent major defects in *Lats2*^iΔEC^ mutants.

At the mechanistic level, our results link LATS2 to EndMT, SRF and the regulation of exocytosis. EndMT is a physiological process during embryonic heart development where it mediates the conversion of ECs into valve interstitial cells but also into cardiac pericytes and vascular SMCs^35^. By contrast, EndMT in the adult organism refers typically to the pathogenic acquisition of a mesenchymal gene signature by ECs in atherosclerosis, hypertension and some other cardiovascular diseases^36^. SRF is a critical regulator of actin polymerization, which, together with myocardin family cofactors, controls the expression of effectors of actin dynamics and, thereby, myofibroblast differentiation and tissue fibrosis in multiple organs^37,38^. Cooperation between YAP1/TAZ and SRF was reported for the differentiation of vascular SMCs but also in certain cancer cells and cancer-associated fibroblasts^39–41^. However, it was also shown that the crosstalk between MRTF-SRF and YAP1/TAZ is often indirect and mediated by cytoskeletal dynamics and cell shape changes^40,42^. Although the exact nature of the link between SRF and Hippo pathway components in the bone endothelium requires further investigation, our findings show that SRF is required for YAP1/TAZ-induced EndMT, which involves marked changes in EC morphology, gene expression and vascular architecture. These alterations in ECs, through increased expression of matrix molecules and other secreted factors, affect other cell types, most likely mesenchymal mpMSCs/Osteo-CAR cells, resulting in fibrosis and osteosclerotic lesions. Furthermore, the identification of vascular defects in the ThPO and MPL models of myelofibrosis raises the possibility that EndMT, increased biological activity of YAP1/TAZ and SRF-mediated changes in gene expression, but also EC cytoskeletal function, might contribute to fibrosis. These findings are consistent with an earlier study reporting EndMT in ECs of small vessels in the BM and spleen of the JAK2-V617F knock-in mouse model but also in patients with primary myelofibrosis^14^. Although the molecular interactions linking diseased hematopoietic cells and blood vessels remain to be unraveled, TGFβ family transforming growth factors were shown to be upregulated in megakaryocytes but also in monocytes/macrophages from patients with myelofibrosis. Inhibition of TGFβ signaling ameliorates fibrosis in vitro and in mouse models of myelofibrosis^43,44^. TGFβ signaling is also a potent inducer of EndMT, has been linked to the loss (rarefaction) of microvessels and can potentiate MRTF-SRF signaling^38,45,46^. Future studies will have to address the role of TGFβ and other molecular regulators and provide further insight into the role of blood vessels in animal models of myelofibrosis and in human patients.

## Methods

### Animal models

For the inducible and EC-specific inactivation of *Lats2* in mice, we bred *Lats2*^lox/lox^ conditional mutants to *Cdh5(PAC)-CreERT2* transgenic mice^21^ to generate Cre-positive EC-specific *Lats2*^iΔEC^ mutants. *Cdh5(PAC)-mT/nG* transgenic reporter mice^21^ were introduced into the *Lats2*^iΔEC^ mutant background and corresponding controls. Mice carrying *loxP*-flanked *Yap1* and *Taz (Wwtr1)* alleles^47^ were also bred to the *Cdh5(PAC)-CreERT2* line to generate EC-specific *Yap1*^lox/lox^ and/or *Taz*^lox/lox^ conditional mutants^21^. To generate EC-specific constitutive-active Yap1 knock-in (Yap1-KI), mutated Yap1 amino acid 112 serine to alanine (Yap1^S112A^) mice^21^ were bred with *Cdh5(PAC)-CreERT2*. Using *Srf*^lox/lox^ mice with a similar breeding strategy, we generated inducible and EC-specific *Srf*^iΔEC^ mutant^48^ and *Lats2 Srf* double loss-of-function mutants (*Lats2 Srf*^iΔEC^). In all these experiments, Cre-negative littermates subjected to the same tamoxifen administration regime were used as controls. For inducible Cre*-*mediated recombination, mice received daily intraperitoneal injections of 1 mg of tamoxifen for 5 d at the age of 4–5 weeks. For the characterization of BMSCs in the metaphysis, *Gli1-CreERT2* (ref. ^49^) transgenic mice were interbred with Gt(Rosa26) ACTB-tdTomato-EGFP reporter mice^50^. These mice received 1 mg of tamoxifen for 3 d at 8 weeks of age and were analyzed 4 weeks later. Tamoxifen stocks were prepared as described previously^21^. Mice were backcrossed and maintained in a C57/BL6 genetic background, and both sexes were used for analysis.

For the ThPO model of bone fibrosis, wild-type (WT) cKit^+^-enriched cells were transduced with ThPO or empty vector (EV) control lentivirus and transplanted into lethally irradiated (split-dose) B6.Cg-Tg(Ly6a-EGFP)G5Dzk/J (*n* = 5 animals per group) and B6.SJL recipient mice (*n* = 3–4 mice per group). Blood was periodically collected via submandibular bleeds into microtainer tubes (Becton Dickinson), and blood counts were performed on a Horiba SciI Vet abc Plus hematology system. Transplantation of MPL^W515L^-expressing and matching control hematopoietic cells into sub-lethally irradiated mice was conducted as described previously^51^. Mice were euthanized for analysis when they displayed signs of fibrosis, as indicated by the dropping of hemoglobin levels or weight loss according to defined humane endpoints. Femurs were collected for imaging.

All animal experiments were performed according to institutional guidelines and laws, approved by local animal ethical committees and conducted at the Max Planck Institute for Molecular Biomedicine with necessary permissions (02.04.2015.A185, 84-02.04.2016.A160, 81-02.04.2017.A238 and 81-02.04.2019.A164) granted by the Landesamt für Natur, Umwelt und Verbraucherschutz of North Rhine-Westphalia, Germany. ThPO and MPL bone fibrosis experiments were conducted according to protocols approved by the Central Animal Committee (Centrale Commissie Dierproeven (CCD), The Netherlands) in accordance with legislation in The Netherlands (approval no. AVD1010020173387).

### Sample processing and immunostaining

Mice were euthanized, and long bones (femur and tibia) were harvested and fixed immediately in ice-cold 2% paraformaldehyde (PFA) for 6–8 h under gentle agitation. Bones were decalcified in 0.5 M EDTA for 48–72 h at 4 °C under gentle shaking agitation, which was followed by overnight incubation in cryopreservation solution (20% sucrose, 2% PVP) and embedding in bone embedding medium (8% gelatine, 20% sucrose, 2% PVP). Samples were stored overnight at −80 °C. Then, 80–100-μm-thick cryosections were prepared for immunofluorescence staining.

Bone immunostaining was performed as described previously. Bone sections were washed in ice-cold PBS and permeabilized with ice-cold 0.3% Triton X-100 in PBS for 10 min at room temperature (RT). Samples were incubated in blocking solution (5% heat-inactivated donkey serum in 0.3% Triton X-100) for 30 min at RT. Primary antibodies: rat monoclonal anti-endomucin (V.7C7) (Santa Cruz Biotechnology, sc-65495, 1:100 dilution), goat polyclonal anti-CD31 (R&D Systems, AF3628, 1:100 dilution), rabbit polyclonal anti-Fabp5 (Lifespan Biosciences, C312991, 1:100 dilution), goat anti-PDGFRβ (R&D Systems, AF1042, 1:100 dilution), mouse monoclonal alpha-smooth muscle actin-Cy3 (Sigma-Aldrich, C6198, 1:200 dilution), mouse monoclonal alpha-smooth muscle actin-eFluor660 (eBioscience, 50-9760-82, 1:200 dilution), rabbit monoclonal anti-Yap1/Taz (D24E4, Cell Signaling Technology, 8418, 1:100 dilution), rabbit monoclonal anti-vimentin (D21H3, Cell Signaling Technology, 5741, 1:100 dilution), rabbit polyclonal anti-Tagln (Sm22a, Abcam, ab14106, 1:100 dilution), rabbit polyclonal anti-fibronectin (Fn1, Sigma-Aldrich, F3648, 1:100 dilution), rabbit polyclonal anti-thrombospondin 1 (Thbs1, Abcam, ab85762, 1:100 dilution), rabbit polyclonal anti-Angpt2 (Abcam, ab8452, 1:100 dilution), rabbit polyclonal anti-Cav1 (Cell Signaling Technology, 3238, 1:100 dilution), rabbit polyclonal anti-Col4 (AbD Serotec, 2150-1470, 1:100 dilution), rabbit-polyclonal anti-Osterix (Abcam, ab22552, 1:300 dilution), goat polyclonal anti-Mmp9 (R&D Systems, AF909, 1:200 dilution), rabbit polyclonal anti-Col-I (Millipore, AB765P, 1:100 dilution), rabbit polyclonal anti-Col-X (Abcam, ab58632, 1:100 dilution), rabbit polyclonal anti-LATS2 (GeneTex, GTX87529, 1:50 dilution), rabbit polyclonal anti-Aggrecan (Milipore, AB1031, 1:100 dilution), rabbit anti-vATPaseB1/B2 (Abcam, 200839, 1:100 dilution), rat anti-mouse Ter119 (BD Biosciences, 553673, 1:200 dilution), rat anti-mouse B220 (BD Biosciences, 553090, 1:200 dilution), rat-monoclonal anti-active Itgb1 (BD Biosciences, 553715, 1:500 dilution), rabbit polyclonal anti-FAK (pY397) (Thermo Fisher Scientific, 44-624G, 1:50 dilution), rabbit monoclonal anti-Runx2 (Abcam, ab192256, 1:200 dilution) or rat monoclonal anti-Srf (from Alfred Nordheim, 1:50 dilution) were diluted in 5% donkey serum mixed PBS and incubated overnight at 4 °C. Next, slides were washed 3–5 times in PBS in 5–10-min intervals. Species-specific Alexa Fluor secondary antibodies Alexa Fluor 488 (Thermo Fisher Scientific, A21208), Alexa Fluor 546 (Thermo Fisher Scientific, A11056), Alexa Fluor 594 (Thermo Fisher Scientific, A21209), Alexa Fluor 647 (Thermo Fisher Scientific, A31573 or A21447) or Phalloidin-488 (Invitrogen, A12379) diluted 1:100 in PBS were added and incubated for 2–3 h at RT. Immunostained bone sections were imaged with a Leica SP8 confocal microscope.

### Vascular permeability assay

To conduct in vivo vascular permeability tests on adult mice, 100 µl of 1 mg of Dextran Texas Red (Thermo Fisher Scientific, D1830, 70,000 MW) was injected into the tail vein and left to circulate for 15 min. Femurs were removed from euthanized mice, immediately dropped in ice-cold 4% PFA and fixed overnight. Fluorescence microscopy was used to capture images of the femur without any signal enhancement.

### Histological staining methods

For Safranin O staining, long bones from Lats2^iΔEC^ mutants and control animals were harvested, fixed and decalcified. The bones were dehydrated and embedded using paraffin wax by stranded histology methods. Bone paraffin blocks were cut at 5 µm, and sections were deparaffinized and hydrated. Then, stain with 0.1% Safranin O for 8 min and wash with water. Next, slides were dehydrated and dried and mounted with non-aqueous mounting medium (Entellan, Sigma-Aldrich).

AZAN trichrome staining after deparaffinization and hydration of the slides. After 5 min at 55 °C in the azocarmin solution, the slides were washed with distilled water. The sections were then immersed in an aniline-ethanol solution for 18 minutes, before they were exposed to 1% acetic acid in ethanol solution for 45 s. Next, rinse with distilled water and then immerse in 5% phosphotungstic acid for 2 h. After that, place in a mixture of Aniline Blue–Orange G for 8 min. Use distilled water to rinse. Next, sections were dehydrated and mounted.

For Reticulin staining, bones were hydrated with distilled water. After a 5-min addition of potassium permanganate solution, distilled water was used to rinse. The next solutions—oxalic acid, silver nitrate, gold chloride and sodium thiosulfate—were added for 2 min and then individually washed with distilled water. Sections were dehydrated and mounted.

### Calcein Green and Alizarin Red double labeling assay

For in vivo double labeling with Calcein Green and Alizarin Red, 10-week-old control and *Lats2*^iΔEC^ mutant mice were injected intraperitoneally with 100 µl of Calcein Green (40 mg kg^−1^; Sigma-Aldrich, C0875) and 100 µl of Alizarin Red (40 mg kg^−1^; Sigma-Aldrich, A3882) 1 week later. Mice were euthanized at 12 weeks, and femurs were dissected and immediately placed in ice-cold 4% PFA for overnight fixation. Next, samples were cryopreserved, embedded in bone embedding media and kept at −80 °C overnight. Bones were cryosectioned at 30-µm thickness, stained for 30 min with DAPI and then washed twice with washing buffer before imaging by confocal fluorescence microscopy.

### µCT analysis

Femurs were fixed in 4% PFA overnight at 4 °C and subjected to µCT analysis by Scanco Medical AG. In the whole three-dimensional evaluation of the trabecular structure, distal section of femur, a voxel size of 5 mm was used. We evaluated 833 slices covering 4.988 mm in height for each sample.

### Fluorescence-activated cell sorting

Single-cell suspensions were prepared from bone and spleen as described above. Primary antibody lineage cell detection cocktail-biotin (Miltenyi Biotec, 130-092-613), rat monoclonal anti-Ly6A-PE-cy7 (BD Pharmingen, clone D7, 558162), rat monoclonal anti-CD117-APC (BD Pharmingen, clone 2B8, 553356), hamster monoclonal anti-CD3e-FITC (eBioscience, clone 145-2C11, 11-0031), rat monoclonal anti-B220-APC (Invitrogen, RA3-6B2, RM2605), rat monoclonal anti-CD11b-APC (BioLegend, M1/70, 101212), rat monoclonal anti-Ly-6G(Gr-1)-BV605 (BioLegend, RB6-8C5, 108440), rat monoclonal anti-CD45-PE (BD Pharmingen, 30-F11, 553081), rat monoclonal anti-TER-119-APC (eBioscience, 17-5921) or rat monoclonal anti-CD41-BV605 (BioLegend, MWReg30, 133921) were diluted in fluorescence-activated cell sorting (FACS) buffer (2% FCS, 0.5% BSA in PBS solution) and incubated with cells on ice for 40 min. Cells were washed 2–3 times in FACS buffer and incubated with secondary antibodies for 40 min. Cells were washed two further times and used for flow cytometry. Cells were resuspended in FACS buffer supplemented with 1 mg ml^−1^ DAPI to allow exclusion of non-viable cells when required. Cell sorting was performed on a FACSAriaIIu cell sorter (BD Biosciences). Cell analysis was performed using FlowJo software.

### BMSC in vitro culture and quantitative PCR

BMSCs were isolated from metaphysis of WT mice and cultured as described previously^52^. For agonist-induced BMSC differentiation assay, cells were serum starved for overnight and then stimulated with agonist TGF-β1, Endothelin-1 (ET-1) or Adrenomedullin (ADM) for 24 h and analyzed for quantitative PCR. Next, total RNA was isolated from these cells using an RNA Plus Mini Kit (Qiagen, 74134) according to the manufacturer’s instructions. RNA was reverse transcribed using the an iScript cDNA Synthesis Kit (Bio-Rad, 1708890). Quantitative PCR was carried out using gene-specific TaqMan probes Eukaryotic *18S rRNA* (4319413E), Acta*2* (Mm00725412_s1), *Tagln* (Mm00441661_g1) and *Fn1* (Mm01256744_m1) (Thermo Fisher Scientific) using a C1000 Touch thermal cycler (Bio-Rad).

### Electron microscopy

Femurs were removed and directly cut into half in fixative. For ultrastructural analysis, fixative comprised 2% glutaraldehyde, 2% PFA, 20 mM CaCl_2_, 20 mM MgCl_2_ in 0.1 M cacodylate buffer, pH 7.4. Fixed material was post-fixed in 1% osmium tetroxide containing 1.5% potassium ferrocyanide, dehydrated, including uranyl-en-bloc staining, and embedded stepwise in epon. Ultramicrotomy was performed until reaching the area of interest, where 60-nm ultra-thin sections were collected on formvar-coated 1 slot copper grids. Sections were further stained with lead citrate and finally analyzed with a transmission electron microscope (Tecnai-12-biotwin, Thermo Fisher Scientific).

### Bone stromal cells preparation for scRNA-seq

For scRNA-seq analysis, femur and tibia were harvested from *Lats2*^iΔEC^ mutant and control mice and cleaned from attached surrounding tissue before the metaphysis region was dissected and collected in digestion enzyme solution (Collagenase type I and type IV, 2 mg ml^−1^). Next, bones were crushed using mortar and pestle. Samples were digested for 45 min at 37 °C under gentle agitation. Digested samples were transferred to 70-μm strainers in 50-ml tubes to obtain a single-cell suspension, which was resuspended in blocking solution (1% BSA, 1 mM EDTA in PBS without Ca^2+^/Mg^2+^), centrifuged at 300*g* for 5 min, washed 2–3 times with ice-cold blocking solution and filtered through 50-μm strainers. Pellets were resuspended in respective volume of blocking solution. Single-cell suspensions were subjected to lineage depletion using a lineage cell depletion kit (MACS, 130-090-858) following the manufacturer’s instructions. Next, lineage-negative (Lin^−^) cells were depleted by CD45 and CD117 using microbeads (MACS, 130-052-301 and 130-091-224) from Lin^−^ bone cells to enrich bone stromal cells. The remaining cells were resuspended to final concentration of 10^6^ cells per milliliter in 0.05% BSA in PBS, examined by microscopy and used for scRNA-seq.

Single-cell suspensions were subjected to droplet-based scRNA-seq. Single cells were encapsulated into emulsion droplets using the Chromium controller (10x Genomics). scRNA-seq libraries were prepared using a Chromium Single Cell 3′ Reagent Kit (V3) (10x Genomics, PN-10000075) according to the manufacturer’s protocol. scRNA-seq libraries were evaluated and quantified by an Agilent Bioanalyzer using a High Sensitivity DNA Kit (cat. no. 5067-4626) and a Qubit (Thermo Fisher Scientific, Q32851). Individual libraries were diluted to 4 nM and pooled for sequencing. Pooled libraries were sequenced by using a High Output Kit (150 cycles) (Illumina, TG-160-2002) with a NextSeq 500 sequencer (Illumina).

### scRNA-seq data analysis

Detailed settings for the analysis steps mentioned below can be found in the accompanying GitLab repository (see ‘Data availability’ section) and in Supplementary Table 1. Default parameters were used, if not detailed otherwise, there or in the text below.

#### Read data quality control and mapping

Illumina sequencing results were demultiplexed and converted to FASTQ format using Illumina bcl2fastq software (version 2.20.0.422). Raw reads were processed using cutadapt^53^ (version 3.5). Pre-processed read data were aligned to the mouse reference genome (version GRCm39) with GENCODE M26 annotation^54^ extended by adding sequences for CreERT2 and Rosa26-mTmG and counted with STARsolo^55^ (version 2.7.10a) using its electron microscopy implementation and GeneFull_Ex50pAS read counting. The official 10x Genomics barcode whitelist^56^ was used.

#### Data filtering and visualization

Pre-processing of the feature-count-matrix output by STARsolo was largely performed within the Python (version 3.8.10)-based scanpy (version 1.9.1) ecosystem^57^. Exceptions are functionalities that are only available for R (ref. ^58^) (version 4.2.1). These include: sequencing depth normalization using scran (version 1.24.1)^59^, doublet detection using scDblFinder (version 1.11.4)^60^ and calculation of DEGs using DESeq2 (version 1.36)^61^. All visualization was done using matplotlib (version 3.5.3)^62^ functions. Barcodes with fewer than 1,000 unique molecular identifiers (UMIs) or fewer than 800 features (>0 counts per feature) detected were discarded. Similarly, only barcodes with less than 15% of counts stemming from mitotic genes or 5% from hemoglobin genes were retained. Furthermore, barcodes were filtered for a complexity value above 0.8, where complexity is defined as the log_10_-transformed number of expressed genes divided by the log_10_-transformed number of total counts per barcode. Additionally, only genes with at least 10 counts total from at least 10 distinct barcodes were kept. scran normalization was applied using min.size = 100 and min.mean = 0.1 in the quickCluster and computeSumFactors functions, respectively. The separate samples were merged into one AnnData (version 0.8.0) object; 4,000 highly variable genes were calculated from the normalized matrix; and the matrix was scaled to unit variance and zero mean, capping values at 10. The 50 first principal components were used for integration using Harmony-Py^63^ (version 0.0.9, Python implementation by Kamil Slowikowski) with max_iter_harmony = 1,000, max_iter_kmeans = 1,000, epsilon_cluster = 1 × 10^−6^ and epsilon_harmony = 1 × 10^−5^. Uniform manifold approximation and projection (UMAP) dimensionality reduction^64^ and Leiden clustering^65^ at a resolution of 1 were performed. The resulting 30 clusters, the uncorrected principal component analysis (PCA) embedding and the raw count and normalized matrices were used for scDblFinder doublet prediction. Barcode clusters were annotated with the broad cell identity that they most likely represent based on marker genes provided in Supplementary Table 1. Undesired populations, such as myeloid or lymphoid cells; clusters of barcodes that, either manually based on gene expression or by scDblFinder, could be classified as consisting of more than 20% doublets; and very small clusters (<100 cells) were removed. All remaining 20,383 barcodes will hereafter be referred to as cells.

For each of the four main groups of broad cell identities defined previously, we created subsets and performed scaling, PCA calculation, harmony-py integration, UMAP visualization and Leiden clustering anew as before, to detect more fine-grained sub-cell-type identities. Details on the settings used in each subclustering and marker genes used for annotation can be found in Supplementary Table 1. Some subclustering revealed small cell clusters that could not be identified based on their expression and were, therefore, removed. The subset was then visualized in a second passthrough (indicated in Supplementary Table 1) with the same settings as the first one. Afterwards, either all subsets (20,184 cells) or only the mp-MSC and SMC subsets were merged again for comprehensive visualization and analysis.

Pseudotime analysis was performed using slingshot^66^ (version 2.6.0). We defined the mpMSC cluster as starting and the Myh11 SMC cluster as the end node of the trajectory. Cells were binned into 21 bins of equal cell numbers along the pseudotime trajectory. Gene-to-gene Pearson correlation of normalized and z-scaled gene expression counts across the ordered pseudotime bins was calculated for the top 8,000 highly variable genes. Correlations with a correlation coefficient above 0.5 were kept, and the data were transformed into an igraph (Python igraph 0.10.4)^67^ network with genes representing nodes and their mutual correlation coefficients representing the edge weights. Leiden clustering was applied with a resolution parameter of 1.

Differential gene expression (DGE) analysis was performed using DESeq2 on pseudobulk expression matrices. When the expression of genes in one cluster was to be compared to several other clusters, all pairwise comparisons were calculated, and then the mean log_2_fold change (FC) of these comparisons was output as log_2_FC, and the minimum *P* value (alpha = 0.01) calculated from the respective adjusted *P* values using scipy.stats’s combine_pvalues function (version 1.9.1)^68^ was used as ‘adjusted *P* value’ of the upregulation of gene expression in the cluster of interest. It was also counted in how many comparisons the overexpression in the cell cluster of interest compared to each other one was above a log_2_FC threshold of 2. If not mentioned otherwise, only genes expressed in at least 20% of cells of the respective group in either condition were kept.

Radial expression plots were created by calculating DGE (using DESeq2) between the control and conditional knockout (cKO) conditions for each cell identity presented in the respective graph. Only genes expressed in at least 20% of cells of the respective group in either condition were kept. Each quadrant represents genes with upregulated expression in the respective condition and cell type. For each quadrant, all log_2_FC values below 0 were set to 0, and the accompanying adjusted *P* values were set to 1. Then, genes were plotted in a scatterplot by jittering points on the *x* axis, representing their log_2_FC on the *y* axis and the adjusted *P* value of the expression difference as the point color. This means that, per cell population, all genes present in the analysis are represented in each quadrant, showing which of these are upregulated in the respective condition and cell type. The main goal of this plot is to show in which condition and cell types most genes are differentially expressed.

#### Additional computational analyses

Differential abundance of cell neighborhoods was quantified using milopy (version 0.1.0)^69^ with alpha = 0.01. Potentially regulating transcription factors for selected gene sets were calculated using the iRegulon^70^ plugin to Cytoscape (version 3.9.1)^71^ with the 6K Motif collection, looking 500 bp upstream of provided genes in seven species. PAGA^28^ was applied to the data with default settings via scanpy’s implementation.

#### Predicting LRIs

To determine possible LRIs, a permutation-based approach similar to the one introduced in CellPhoneDB^72^ was used. The normalized count matrix was used as a basis for 10,000 cell type label permutations where cell type labels were randomly re-assigned. Ligand–receptor (LR) gene identity and interaction pairs were taken from the consensus database of LIANA^73^, excluding bidirectional annotations and genes expressed in less than 10% of cells. For each LR interaction, first the mean expression of the ligand (Lmean) and receptor (Rmean) were calculated for each cell type in each condition, and then an interaction score (LRIScore) was calculated for all possible cell type interactions per condition separately as the product of Lmean and Rmean. This approach differs from the methodology employed by CellPhoneDB in that our approach uses the product instead of the mean value of ligand and receptor mean expression. This is intended to implicitly penalize interactions where one or both partners are lowly expressed. Based on the number of permutation results matching or exceeding the observed value, values of *P* were calculated for each interaction. Values of *P* calculated in this test are approximations of *P* but will be referred to as *P* values nonetheless. Multiple testing correction was performed using the Benjamini–Hochberg^74^ implementation in the statsmodels (version 0.13.1)^75^ function multipletests on all *P* values. These corrected *P* values will be referenced as adjusted *P* values. Additionally, using the created background distribution, the z-score of the original LRIScore was determined.

In the same fashion as for cell type labels, condition labels (control/cKO) were permuted an equal number of times as cell type labels and a difference score between the LRI scores of the two conditions were calculated for each cell type interaction as:

$${LRIDif}{f}_{{score}}=\frac{\log 2({Lmea}{n}_{{cKO}}/{Lmea}{n}_{{control}})+\log 2({Rmea}{n}_{{cKO}}/{Rmea}{n}_{{control}})}{2}$$

This scoring is similar to a previously proposed perturbation score^76^, but the mean and not the product of the absolute log_2_FC was used. This was done to emphasize coordinated upregulation or downregulation of ligand and receptor instead of just the overall magnitude of change. *P* values and adjusted *P* values were calculated from these scores in a manner sensitive to the direction of change and used to assess differences in LRI scores between conditions. This is similar to a very recently published approach^77^.

This procedure was done once for a dataset, where, of all ECs, only mpECs were kept, and major cell type labels were used for all other cells. Another analysis focused on mpECs, mpMSCs, bmMSCs, Myh11^+^ and Myh11^−^ SMCs compared to all other major cell types as background, but only the results for the mentioned subtypes were visualized.

### Statistics and reproducibility

Images were analyzed, quantified and processed using Volocity (PerkinElmer), Fiji (ImageJ) and Adobe Photoshop and Illustrator software 2020.

Statistical analysis was performed using GraphPad Prism 9 software or the R statistical environment (http://r-project.org). All data are presented as mean values ± s.e.m. unless indicated otherwise. Comparisons between two groups were performed with unpaired two-tailed Mann–Whitney test. Comparisons between more than two groups were made by Tukey multiple comparison test to determine statistical significance. *P* < 0.05 was considered significant unless stated otherwise. For DGE (Figs. 5d and 6a,b and Extended Data Fig. 7g): DESeq2 (internally uses Wald test statistics) yielding false discovery rate (FDR)-corrected *P* values (*P*.adj). Extended Data Fig. 9a: genes filtered based on pairwise comparisons between mp-ECs and all other EC subtypes using DESeq2 yielding FDR-corrected *P* values (*P*.adj) combined across comparisons using the Fisher method implemented in scipy’s combine_p_values function. LR pairings and the significant interactions between cell types of interest (Fig. 6g,h) were analyzed with a permutation test (one-sided) with Benjamini–Hochberg-corrected *P* values (*P*.adj). Extended Data Fig. 9a,c,d used a permutation test (one-sided) with Benjamini–Hochberg-corrected *P* values (*P*.adj). Four biological independent samples were used in Figs. 1e,f, 2i,j and 5f, and three biological independent samples were used in Figs. 3a,e and 4c and Extended Data Figs. 1c–e, 2b–g, 3b, 4f–h, 5f,g,i, 6a and 8b–d. Sample numbers are indicated in the figure legends and were chosen based on experience from previous experiments. Reproducibility was ensured by more than three independent experiments (aside from scRNA-seq). No statistical method was used to predetermine sample size, and no animals were excluded from analysis.

### Reporting summary

Further information on research design is available in the Nature Portfolio Reporting Summary linked to this article.

### Integrated supplementary information

Extended Data Fig. 1 Loss of endothelial Lats2 induces vascular defects in bone. **a**. *Lats2*^iΔEC^ mutant mice look normal and body weight is comparable to control littermates (n = 6). Data shown as mean ± SEM. P values, Mann–Whitney test (two-tailed). **b**. Dot plot for normalised and averaged expression indicates of *Lats1* and *Lats2* expression in the indicated control (Cont) and *Lats2*^iΔEC^ mutant (Mut) EC subpopulations. Z-scaled per row. Note reduction of *Lats2* transcripts and absence of compensatory *Lats1* upregulation. **c**. Reduced LATS2 immunostaining (gray/green) in *Lats2*^iΔEC^ mutant bone endothelium (EMCN, red) (arrowheads). Nuclei, DAPI (blue). **d**. Representative high magnification images of control and *Lats2*^iΔEC^ mutant in *Cdh5-mTnG* reporter background. Femoral metaphysis (mp), bone marrow (bm), growth plate (gp) and transition zone between metaphysis and diaphysis (tz) are indicated. **e**. Representative confocal images showing increase CAV1+ (red), CD31+ (green) and EMCN+ (red) ECs in *Lats2*^iΔEC^ femoral transition zone (tz). Note that CAV1 labels artery in control but microvascular structures in mutant (arrowheads). *n* = independent biological samples.
Source data

Extended Data Fig. 2 Bone defects after loss of Lats2 in ECs. **a**. Tile scan confocal longitudinal views of EMCN and CD31 stained femoral blood vessels. Note enlargement of *Lats2*^iΔEC^ growth plate and metaphysis. Type H capillaries are visible in control but are replaced by CD31+/EMCN- ECs in mutants (arrowheads). Graphs on the right show quantification of ECs in bone marrow (bm) (top) and CD31+/EMCN- capillaries (bottom) (n = 6). Data presented as mean ± SEM. P values, Mann–Whitney test (two-tailed). **b**. Representative images showing expansion of hypertrophic chondrocytes in *Lats2*^iΔEC^ mutants by Safranin O staining and collagen type X (COL-X) immunostaining. **c**. FABP5+ septoclasts and MMP9 expression (arrowheads) at chondro-osseous border near growth plate (gp) are lost in *Lats2*^iΔEC^ mutants. **d, e**. *Lats2*^iΔEC^ femurs show increase in PDGFRβ+ and αSMA+ immunosignals (arrows). αSMA+ in control is largely confined to arterial SMCs (arrowheads) (**d**). Higher magnification images are shown in (**e**). **f**. AZAN trichrome staining of femoral sections showing expansion of the *Lats2*^iΔEC^ growth plate (gp) (left) and ectopic bone (center). Insets on the right show deposition of collagen fibers (arrowheads), which is visible at high magnification. **g**. Representative images showing increased collagen type I (COL-I) immunosignal and reticulin stained fibers in *Lats2*^iΔEC^ mutant sections. *n* = independent biological samples.
Source data

Extended Data Fig. 3 Loss of Lats2 in ECs promotes bone fibrosis. **a**, **b**. Tamoxifen injection scheme (**a**). Tile scan confocal longitudinal view of femurs stained for EMCN (red) and CD31 (green) in 8 and 10-week-old control or *Lats2*^iΔEC^ mutant mice (**b**). Nuclei, DAPI (blue). **c**. Confocal images of αSMA+ (green) cells in 8-week-old and 10-week-old control or *Lats2*^iΔEC^ femoral metaphysis (mp). EMCN (red), DAPI (blue). **d**. Quantification of number and length of vessels columns as well as CD31+/EMCN- area (n = 4). Data presented as mean ± SEM. P values, Mann–Whitney test (two-tailed). **e**. Representative images of control and *Lats2*^iΔEC^ organs in the *Cdh5-mTnG* reporter background. Sections are stained for αSMA, EMCN and YAP1/TAZ, as indicated. Quantification shows that EC number is unchanged in these organs (n = 4). Data shown as mean ± SEM. P values, Mann–Whitney test (two-tailed). *n* = independent biological samples.
Source data

Extended Data Fig. 4 Blood cells and spleen morphology in *Lats2*^iΔEC^ mutants. **a**, **b**. Quantification of white blood cells (WBC), red blood cells (RBC), and platelets (PLT) in peripheral blood (**a**). FACS analysis of Ter119+, B220+, and CD45+ cell frequencies in bone (**b**). **c**, **d**. Representative confocal images of CD41+ megakaryocytes (green) in control and *Lats2*^iΔEC^ femoral sections (**c**). Quantification shows significant reduction of CD41+ cells in the *Lats2*^iΔEC^ metaphysis fibrotic area but not in diaphysis of non-fibrotic area (**d**). **e**. Quantification of B220+ and CD45+ cell frequencies in spleen. **f**. Confocal tile scans of control and *Lats2*^iΔEC^ spleen vasculature labelled with the *Cdh5-mTnG* reporter (left). Images at high magnification show αSMA (green), ECs (*Cdh5-mTnG*, red), and DAPI (blue). White pulp (wp) and red pulp (rp) are indicated. **g**, **h**. Representative confocal images of the spleen showing B220 (green) and Ter119 (red) positive white pulp (wp) and red pulp (rp), respectively. Blood vessels (*Cdh5-mT*, blue) (**g**). Immunostaining of YAP1/TAZ in *Cdh5-mTnG* reporter of control and *Lats2*^iΔEC^ spleen (**h**). Sample number is n = 6 (a, b), n = 4 (d), n = 6 (e). Data are presented as mean ± SEM. P values, Mann–Whitney test (two-tailed). *n* = independent biological samples.
Source data

Extended Data Fig. 5 YAP1/TAZ expression in ThPO-or MPL induced myelofibrosis. **a-c**. Quantification of white blood cells (WBC), red blood cells (RBC) and platelets (PLT) in peripheral blood (**a**), spleen weight (**b**), and number of type H vessel buds (**c**) in control and ThPO gain-of-function bone. (n = 5). Data are presented as mean ± SEM. P values, Mann–Whitney test (two-tailed). **d, e**. Representative confocal images showing increase of αSMA immunosignals in diaphysis (**d**) and reduction of OSX+ cells (**e**) in ThPO femur relative to control. (n = 4). Data are presented as mean ± SEM. P values, Mann–Whitney test (two-tailed). **f**. Representative confocal images showing strong and widespread increase in αSMA (gray) immunostaining together with increased YAP1/TAZ expression ThPO-treated BM. **g**. Experimental scheme of MPL^W515L^-induced BM fibrosis. Representative confocal images (bottom) show αSMA immunostaining in control and MPL^W515L^ (MPL) bone sections. **h**. Confocal images showing reduced EMCN+ (red) vessels and increase of αSMA (green) signal in MPL bone relative to control. Nuclei, DAPI (blue). Graphs on the right show decreased EMCN+ vessels area and expression in MPL bones. (n = 3-4). Data shown as mean ± SEM. P values, Mann–Whitney test (two-tailed). **i**. Immunostaining of YAP1/TAZ (green), EMCN (red), and DAPI (blue) as indicated, in control and MPL bone sections. *n* = independent biological samples.
Source data

Extended Data Fig. 6 Endothelial Hippo signaling in adult bone. **a**. Confocal images showing vATPase+ osteoclasts (red, arrowheads) in *Lats2*^iΔEC^ femur. **b**, **c**. Representative microCT images of cortical bone (cb) in *Lats2*^iΔEC^ and control femur (**b**). Graphs show quantitative analysis of trabecular bone (tb) thickness, tb separation, and cortical bone thickness (**c**). **d**. Representative confocal images and quantification showing slight reduction of femoral OSX+ cells in *Yap1/Taz*^iΔEC^ double mutant relative to littermate control. **e**. Representative µCT images of trabecular bone in control and *Yap1/Taz*^iΔEC^ femur. Quantitative analysis of trabecular (tb) volume (BV/TV, bone volume/total volume) and tb number. Sample numbers n = 5 (b, c), n = 4 (d), n = 5 (e). Data are presented as mean ± SEM. P values, statistical analysis performed using the Mann–Whitney test (two-tailed). *n* = independent biological samples.
Source data

Extended Data Fig. 7 scRNA-sequencing of non-hematopoietic cells from bone. **a-c**. UMAP plot showing color-coded cell clusters of control and *Lats2*^iΔEC^ bone stromal cells (**a**). Markers for main bone stromal cell populations (**b**) and their subclusters (**c**) are shown in dot plots. **d**- **f**. UMAP plot of EC subsets, split by condition (right) (**d**). Bar graph displaying percentage of endothelial cells subtypes in control and *Lats2*^iΔEC^ mutants (**e**). UMAP plot colored by normalized expression of EC subset identity marker genes: *Ramp3* (metaphyseal ECs, mpECs), *Gja4* (arterial ECs, aEC), *Fmo* (arteriole-like ECs, alECs) and *Stab2* (bone marrow ECs, bm) (**f)**. **g**, **h**. Volcano plot of differentially expressed genes (frac. expressed > 0.2 in either condition) between control and mutant condition across all ECs (blue color: log2FC >=1,p.adj <=0.001,) (**g**). Visualisation of selected known Hippo pathway target genes: *Ccn1* (Cyr61), *Ccn2* (Ctgf), *Angpt2* and *Thbs1* in *Lats2*^iΔEC^ mutant and control (**h**).

Extended Data Fig. 8 EndMT in *Lats2*^iΔEC^ bone endothelium. **a**. Confocal images showing strongly elevated Angiopoietin 2 (ANGPT2), Thrombospondin 1 (THBS1), Fibronectin 1 (FN1), Transgelin (TAGLN), and Vimentin (VIM) expression in *Lats2*^iΔEC^ bone sections. (n = 4). Data in graphs are presented as mean ± SEM. P values, statistical analysis performed using the Mann–Whitney test (two-tailed)). **b**. Confocal images showing increased staining for collagen IV (COL IV), the actin cytoskeleton (with Phalloidin), active-integrin β1 and phosphorylated focal adhesion kinase (FAK pY397) in *Lats2*^iΔEC^ mutant relative to control. **c**. Transmission electron micrographs showing increased subendothelial basement membrane and accumulation of ECM (yellow arrowheads) in *Lats2*^iΔEC^ femur. Mutant ECs are rich in actin filaments (red arrowheads). **d**. Confocal images of *Lats2*^iΔEC^ mutant in *R26-dTomato* reporter background showing αSMA staining in dTomato+ ECs. In control samples, αSMA signal is confined to SMCs covering arteries. *n* = independent biological samples.
Source data

Extended Data Fig. 9 Angiocrine signals and EC-BMSC cross-talk. **a**. Ligands showing on average significantly (log2FC >=1,p.adj <=0.05, frac. expressed >=0.1) larger counts in mutant mpECs than in any other EC subidentity and are significantly elevated (see Methods) relative to at least one other EC subpopulation. **b**. Selected ligand-receptor parings of main bone stromal cells, which are increased in *Lats2*^iΔEC^ mutants. **c**. Mean normalized expression counts of selected, significant ligand-receptor interactions (p.adj of LRI score in either condition and LRIDiff Scores <= 0.05, |LRIDiff| >= 1) in mpECS and all other major celltypes. Ligand (left, dark blue bar) and receptor (right, red bar). **d**. Mean normalized expression counts of selected, significant ligand-receptor interactions (p.adj of LRI score in either condition and LRIDiff Scores <= 0.05, LRIDiff >= 1) in mpECS, mpMSCs and SMCs. Ligand (left, dark blue bar) and receptor (right, red bar). **e**. Quantitative PCR (qPCR) shows expression of myofibroblasts markers *Acta2*, *Tagln* and *Fn1* in cultured BMSCs upon treatment with recombinant TGFβ1, endothelin 1 (ET1) or adrenomedullin (ADM). (n = 3). Data in graphs are presented as mean ± SEM. P values, statistical analysis performed using the Mann–Whitney test (two-tailed)). *n* = independent biological samples.
Source data

Extended Data Fig. 10 Lats2 and SRF control EC behavior. **a**, **b**. Representative confocal images of control and *Srf*^iΔEC^ femur show type H (EMCN^high^; CD31^high^) capillaries, Nuclei, DAPI (blue) (**a**) as well as PDGFRβ+ cells (red) and αSMA+ arterial SMCs (green) and EMCN (blue) in the femoral metaphysis (**b**). Graphs in (**b**) show quantification of vessel columns and arteries (n = 6). Data presented as mean ± SEM. P values, Mann–Whitney test (two-tailed). **c**-**e**. Confocal images showing ACAN+ (green) chondrocytes in femoral growth plate (gp) (**c**) and OSX+ cells (green) (**d**). µCT images of trabecular bone of *Srf*^iΔEC^ mutant and control(**e**) and quantitation of trabecular (tb) volume (BV/TV, bone volume/total volume) tb number and gp length (n = 6). Data presented as mean ± SEM. P values, Mann–Whitney test (two-tailed). **f**, **g**. Longitudinal tile scan confocal images showing EMCN+ (red) CD31+ (green) vessels in control, *Lats2*^iΔEC^ and *Lats2 Srf*^iΔEC^ compound mutant femur (**f**). Metaphysis (mp), growth plate (gp, dashed lines) and bone marrow (bm) are indicated. Quantification shows increase in CD31+ EMCN- area in *Lats2*^iΔEC^ metaphysis and rescue by inactivation of *Srf* (**g**) (n = 4). Data presented as mean ± SEM. P values, Tukey multiple comparison test (one-way Anova). **h**. Representative images showing OSX+ cells in control, *Lats2*^iΔEC^, and *Lats2 Srf*^iΔEC^ compound mutant femur. **i, j**. Representative μCT images of femoral trabecular bone (**i**). Quantitative analysis of trabecular bone (tb) thickness and separation (**j**) (n = 4). Data presented as mean ± SEM. P values, Tukey multiple comparison test (one-way Anova). **k**. Proposed model of YAP1/TAZ-induced endothelial-to-mesenchymal transition (EndMT) as a driver of myelofibrosis and osteosclerosis. *n* = independent biological samples. Created with BioRender.com.
Source data

### Supplementary information

Supplementary Fig. 1 and Supplementary Tables 1 and 2.

Reporting Summary

### Source data

Source Data Fig. 1 Statistical source data.

Source Data Fig. 2 Statistical source data.

Source Data Fig. 3 Statistical source data.

Source Data Fig. 4 Statistical source data.

Source Data Fig. 9 Statistical source data.

Source Data Extended Data Fig./Table 1 Statistical source data.

Source Data Extended Data Fig./Table 2 Statistical source data.

Source Data Extended Data Fig./Table 3 Statistical source data.

Source Data Extended Data Fig./Table 4 Statistical source data.

Source Data Extended Data Fig./Table 5 Statistical source data.

Source Data Extended Data Fig./Table 6 Statistical source data.

Source Data Extended Data Fig./Table 8 Statistical source data.

Source Data Extended Data Fig./Table 9 Statistical source data.

Source Data Extended Data Fig./Table 10 Statistical source data.

## Data Availability

The scRNA-seq data generated in this study have been deposited in the Gene Expression Omnibus under accession number GSE225429. The mouse reference genome GRCm39 with GENCODE M26 anotation (https://www.gencodegenes.org/mouse/release_M26.html) was used for mapping the reads in this study. All other relevant data supporting the key findings of this study are available within the article and its Supplementary Information files.

## Code availability

All code used to produce the computational analysis results and figures in this publication are available at https://gitlab.gwdg.de/pmajev/sivaraj_majev_lats2_ecs. The assigned DOI is 10.5281/zenodo.11456485.
