# Supplementary figures and images for: Endothelial LATS2 is a suppressor of bone marrow fibrosis
Source: Nat Cardiovasc Res. 2024 Jul 29;3(8):951–69. doi: 10.1038/s44161-024-00508-x (PMC11324521; doi:10.1038/s44161-024-00508-x)

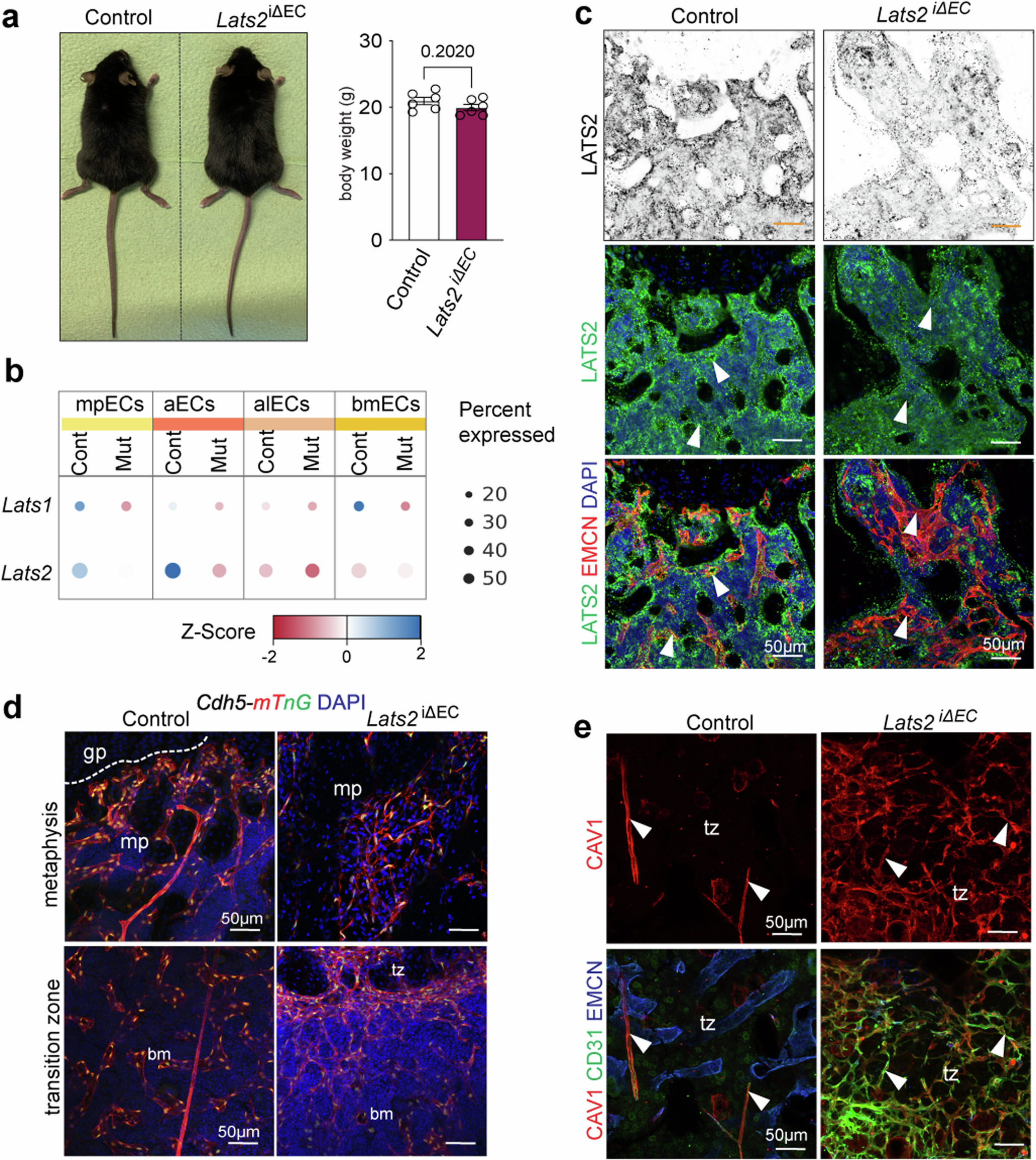

Supplement: Loss of endothelial Lats2 induces vascular defects in bone. — a. Lats2iΔEC mutant mice look normal and body weight is comparable to control littermates (n = 6). Data shown as mean ± SEM. P values, Mann–Whitney test (two-tailed). b. Dot plot for normalised and averaged expression indicates of Lats1 and Lats2 expression in the indicated control (Cont) and Lats2iΔEC mutant (Mut) EC subpopulations. Z-scaled per row. Note reduction of Lats2 transcripts and absence of compensatory Lats1 upregulation. c. Reduced LATS2 immunostaining (gray/green) in Lats2iΔEC mutant bone endothelium (EMCN, red) (arrowheads). Nuclei, DAPI (blue). d. Representative high magnification images of control and Lats2iΔEC mutant in Cdh5-mTnG reporter background. Femoral metaphysis (mp), bone marrow (bm), growth plate (gp) and transition zone between metaphysis and diaphysis (tz) are indicated. e. Representative confocal images showing increase CAV1+ (red), CD31+ (green) and EMCN+ (red) ECs in Lats2iΔEC femoral transition zone (tz). Note that CAV1 labels artery in control but microvascular structures in mutant (arrowheads). n = independent biological samples. Source data [file 44161_2024_508_Fig10_ESM.jpg]

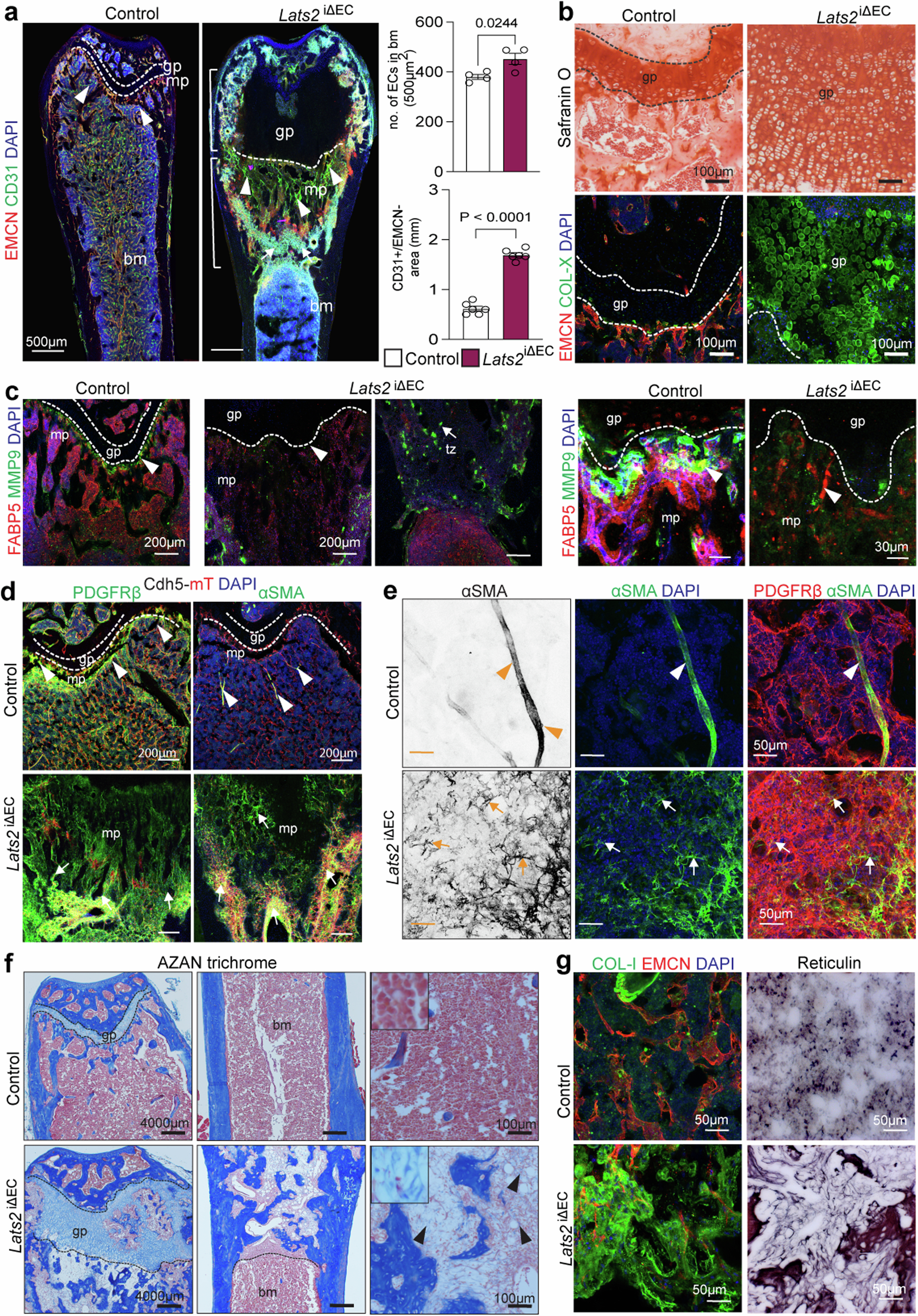

Supplement: Bone defects after loss of Lats2 in ECs. — a. Tile scan confocal longitudinal views of EMCN and CD31 stained femoral blood vessels. Note enlargement of Lats2iΔEC growth plate and metaphysis. Type H capillaries are visible in control but are replaced by CD31+/EMCN- ECs in mutants (arrowheads). Graphs on the right show quantification of ECs in bone marrow (bm) (top) and CD31+/EMCN- capillaries (bottom) (n = 6). Data presented as mean ± SEM. P values, Mann–Whitney test (two-tailed). b. Representative images showing expansion of hypertrophic chondrocytes in Lats2iΔEC mutants by Safranin O staining and collagen type X (COL-X) immunostaining. c. FABP5+ septoclasts and MMP9 expression (arrowheads) at chondro-osseous border near growth plate (gp) are lost in Lats2iΔEC mutants. d, e. Lats2iΔEC femurs show increase in PDGFRβ+ and αSMA+ immunosignals (arrows). αSMA+ in control is largely confined to arterial SMCs (arrowheads) (d). Higher magnification images are shown in (e). f. AZAN trichrome staining of femoral sections showing expansion of the Lats2iΔEC growth plate (gp) (left) and ectopic bone (center). Insets on the right show deposition of collagen fibers (arrowheads), which is visible at high magnification. g. Representative images showing increased collagen type I (COL-I) immunosignal and reticulin stained fibers in Lats2iΔEC mutant sections. n = independent biological samples. Source data [file 44161_2024_508_Fig11_ESM.jpg]

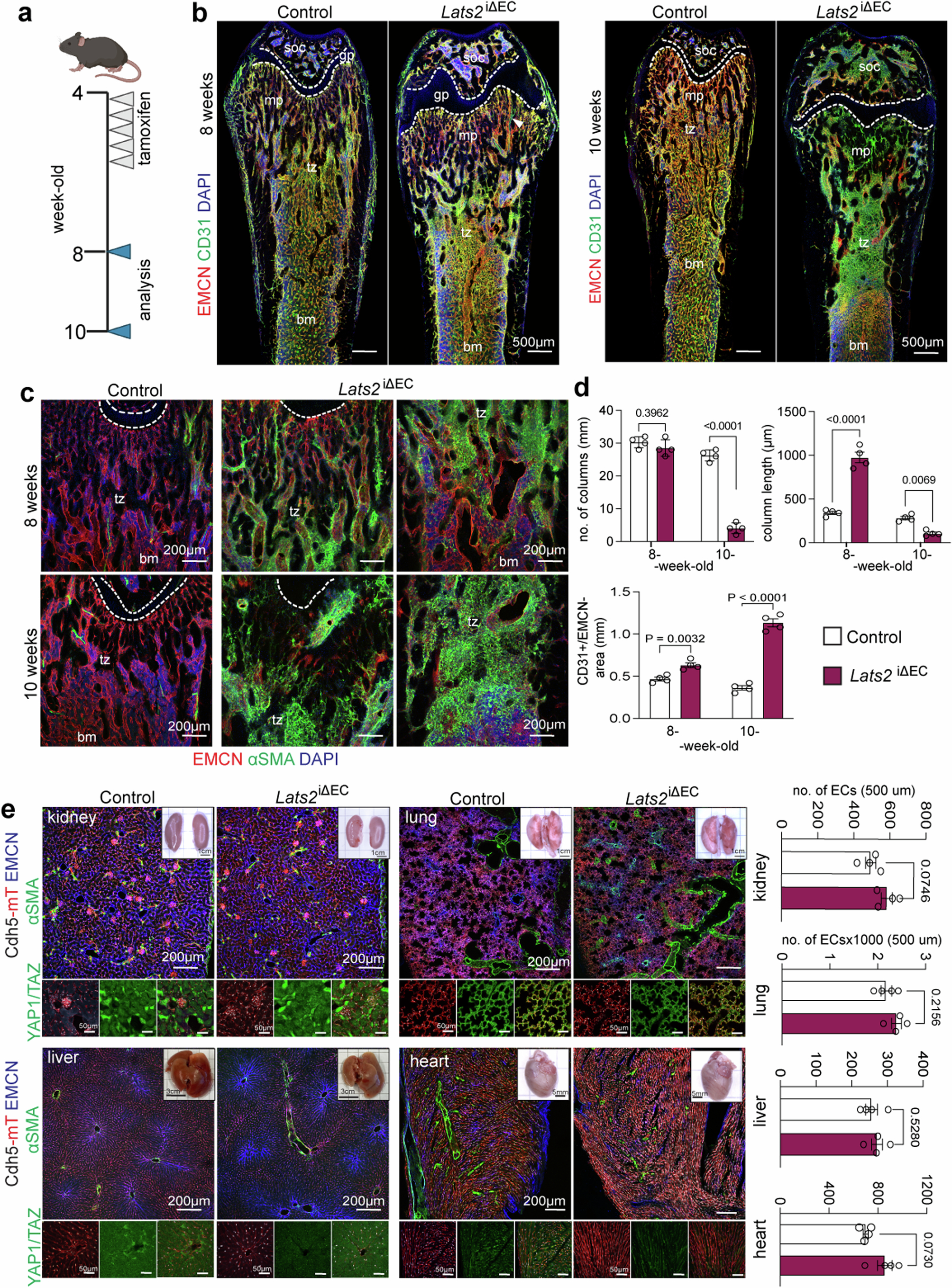

Supplement: Loss of Lats2 in ECs promotes bone fibrosis. — a, b. Tamoxifen injection scheme (a). Tile scan confocal longitudinal view of femurs stained for EMCN (red) and CD31 (green) in 8 and 10-week-old control or Lats2iΔEC mutant mice (b). Nuclei, DAPI (blue). c. Confocal images of αSMA+ (green) cells in 8-week-old and 10-week-old control or Lats2iΔEC femoral metaphysis (mp). EMCN (red), DAPI (blue). d. Quantification of number and length of vessels columns as well as CD31+/EMCN- area (n = 4). Data presented as mean ± SEM. P values, Mann–Whitney test (two-tailed). e. Representative images of control and Lats2iΔEC organs in the Cdh5-mTnG reporter background. Sections are stained for αSMA, EMCN and YAP1/TAZ, as indicated. Quantification shows that EC number is unchanged in these organs (n = 4). Data shown as mean ± SEM. P values, Mann–Whitney test (two-tailed). n = independent biological samples. Source data [file 44161_2024_508_Fig12_ESM.jpg]

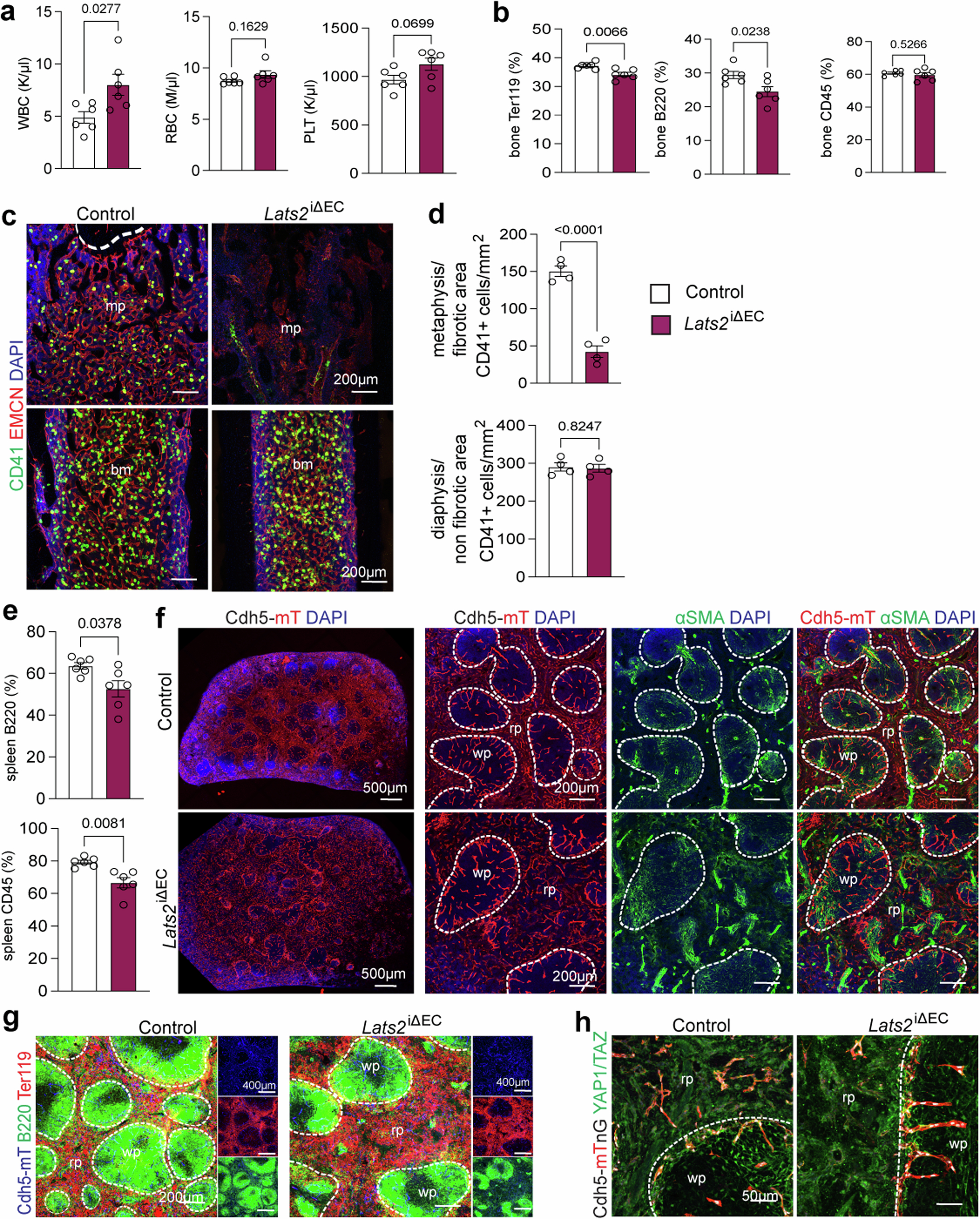

Supplement: Blood cells and spleen morphology in Lats2iΔEC mutants. — a, b. Quantification of white blood cells (WBC), red blood cells (RBC), and platelets (PLT) in peripheral blood (a). FACS analysis of Ter119+, B220+, and CD45+ cell frequencies in bone (b). c, d. Representative confocal images of CD41+ megakaryocytes (green) in control and Lats2iΔEC femoral sections (c). Quantification shows significant reduction of CD41+ cells in the Lats2iΔEC metaphysis fibrotic area but not in diaphysis of non-fibrotic area (d). e. Quantification of B220+ and CD45+ cell frequencies in spleen. f. Confocal tile scans of control and Lats2iΔEC spleen vasculature labelled with the Cdh5-mTnG reporter (left). Images at high magnification show αSMA (green), ECs (Cdh5-mTnG, red), and DAPI (blue). White pulp (wp) and red pulp (rp) are indicated. g, h. Representative confocal images of the spleen showing B220 (green) and Ter119 (red) positive white pulp (wp) and red pulp (rp), respectively. Blood vessels (Cdh5-mT, blue) (g). Immunostaining of YAP1/TAZ in Cdh5-mTnG reporter of control and Lats2iΔEC spleen (h). Sample number is n = 6 (a, b), n = 4 (d), n = 6 (e). Data are presented as mean ± SEM. P values, Mann–Whitney test (two-tailed). n = independent biological samples. Source data [file 44161_2024_508_Fig13_ESM.jpg]

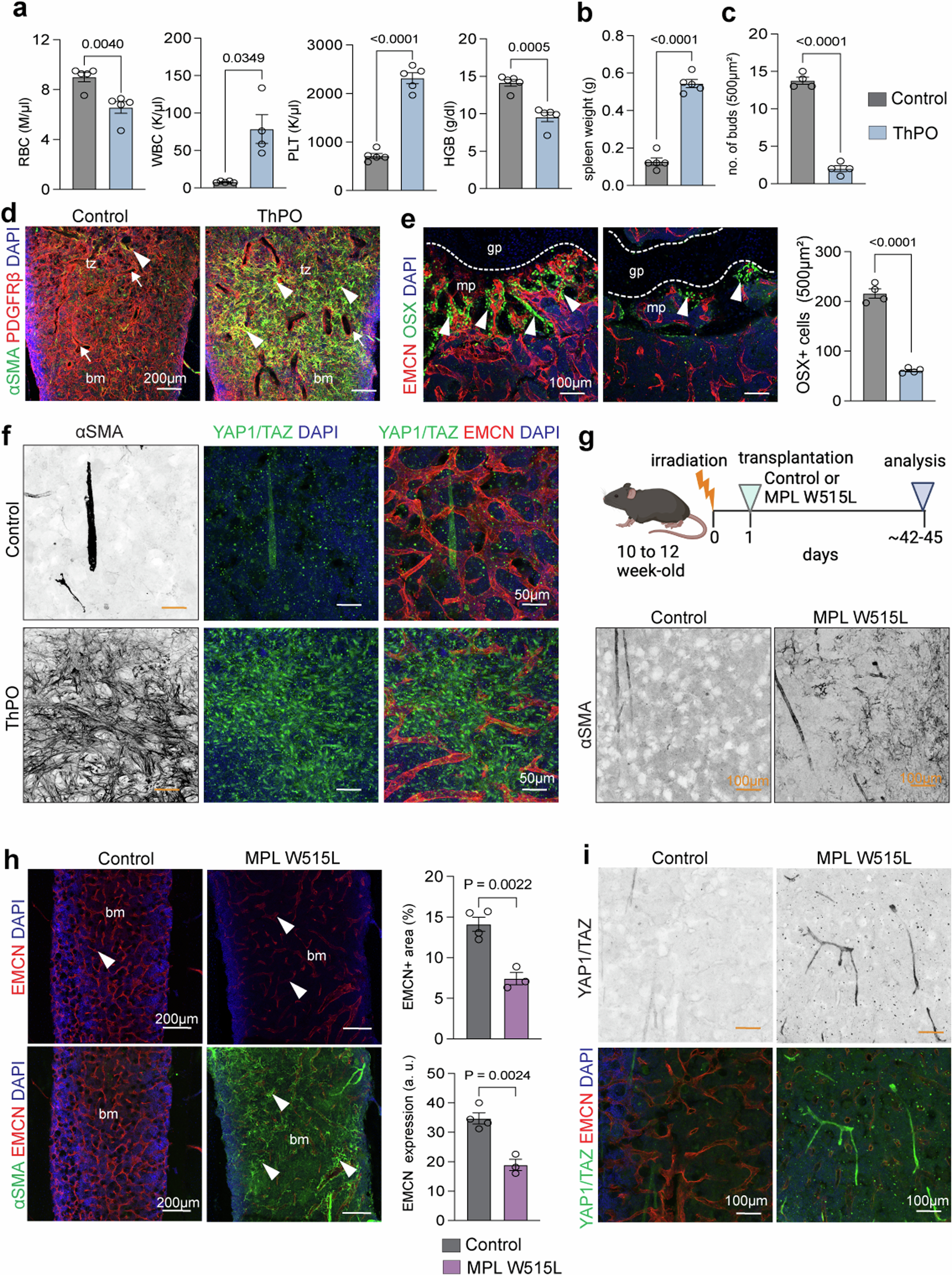

Supplement: YAP1/TAZ expression in ThPO-or MPL induced myelofibrosis. — a-c. Quantification of white blood cells (WBC), red blood cells (RBC) and platelets (PLT) in peripheral blood (a), spleen weight (b), and number of type H vessel buds (c) in control and ThPO gain-of-function bone. (n = 5). Data are presented as mean ± SEM. P values, Mann–Whitney test (two-tailed). d, e. Representative confocal images showing increase of αSMA immunosignals in diaphysis (d) and reduction of OSX+ cells (e) in ThPO femur relative to control. (n = 4). Data are presented as mean ± SEM. P values, Mann–Whitney test (two-tailed). f. Representative confocal images showing strong and widespread increase in αSMA (gray) immunostaining together with increased YAP1/TAZ expression ThPO-treated BM. g. Experimental scheme of MPLW515L-induced BM fibrosis. Representative confocal images (bottom) show αSMA immunostaining in control and MPLW515L (MPL) bone sections. h. Confocal images showing reduced EMCN+ (red) vessels and increase of αSMA (green) signal in MPL bone relative to control. Nuclei, DAPI (blue). Graphs on the right show decreased EMCN+ vessels area and expression in MPL bones. (n = 3-4). Data shown as mean ± SEM. P values, Mann–Whitney test (two-tailed). i. Immunostaining of YAP1/TAZ (green), EMCN (red), and DAPI (blue) as indicated, in control and MPL bone sections. n = independent biological samples. Source data [file 44161_2024_508_Fig14_ESM.jpg]

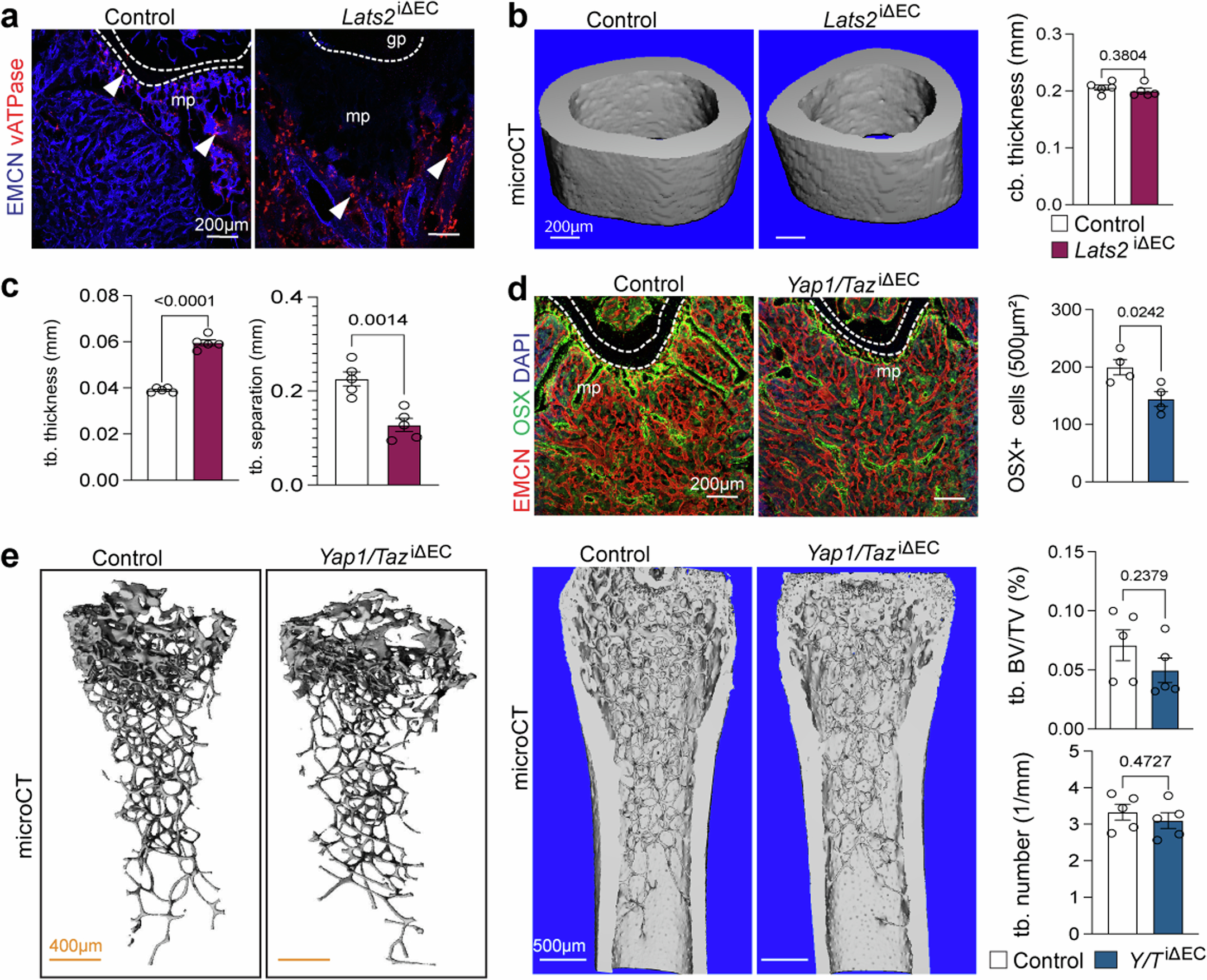

Supplement: Endothelial Hippo signaling in adult bone. — a. Confocal images showing vATPase+ osteoclasts (red, arrowheads) in Lats2iΔEC femur. b, c. Representative microCT images of cortical bone (cb) in Lats2iΔEC and control femur (b). Graphs show quantitative analysis of trabecular bone (tb) thickness, tb separation, and cortical bone thickness (c). d. Representative confocal images and quantification showing slight reduction of femoral OSX+ cells in Yap1/TaziΔEC double mutant relative to littermate control. e. Representative µCT images of trabecular bone in control and Yap1/TaziΔEC femur. Quantitative analysis of trabecular (tb) volume (BV/TV, bone volume/total volume) and tb number. Sample numbers n = 5 (b, c), n = 4 (d), n = 5 (e). Data are presented as mean ± SEM. P values, statistical analysis performed using the Mann–Whitney test (two-tailed). n = independent biological samples. Source data [file 44161_2024_508_Fig15_ESM.jpg]

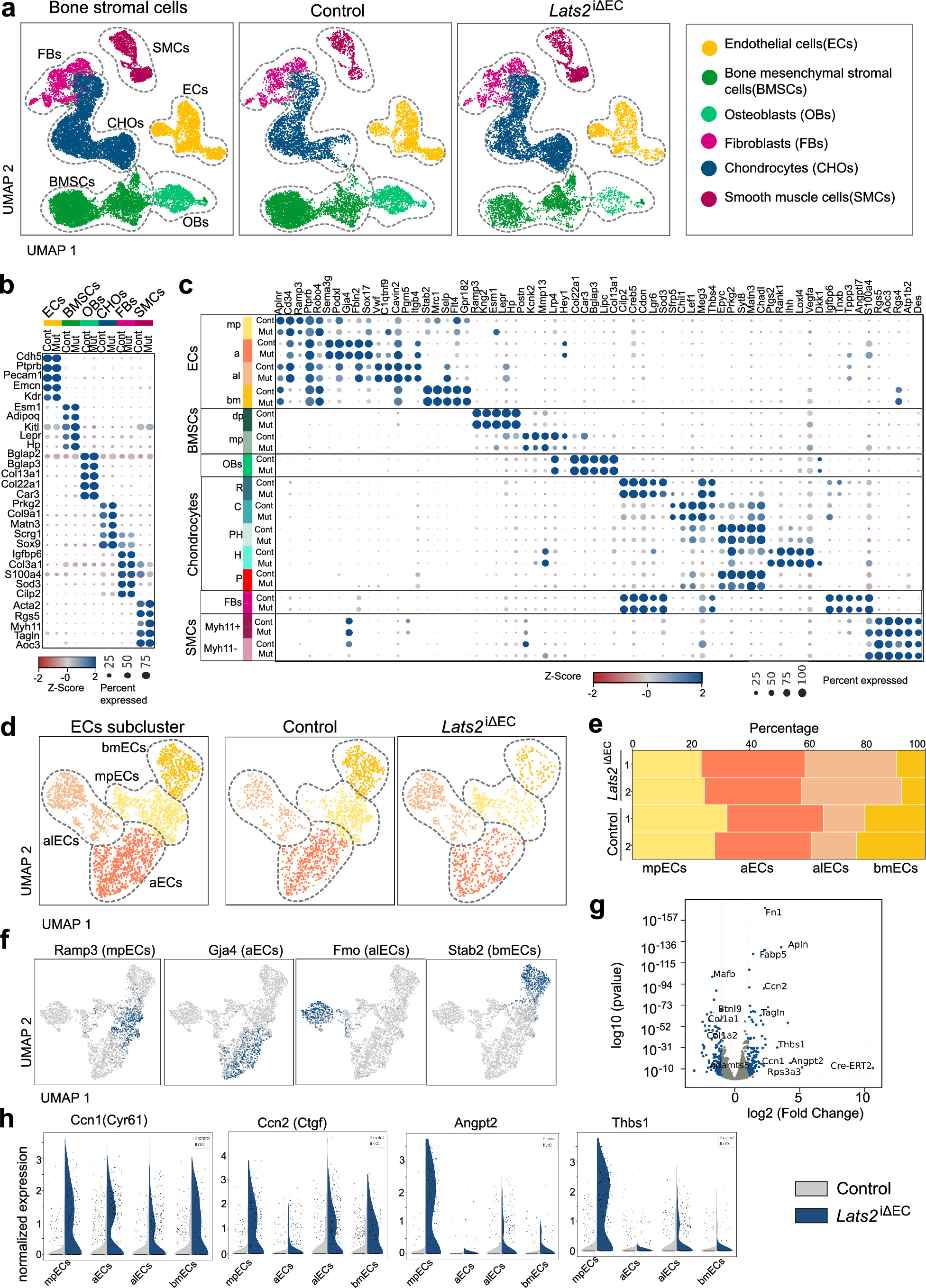

Supplement: scRNA-sequencing of non-hematopoietic cells from bone. — a-c. UMAP plot showing color-coded cell clusters of control and Lats2iΔEC bone stromal cells (a). Markers for main bone stromal cell populations (b) and their subclusters (c) are shown in dot plots. d- f. UMAP plot of EC subsets, split by condition (right) (d). Bar graph displaying percentage of endothelial cells subtypes in control and Lats2iΔEC mutants (e). UMAP plot colored by normalized expression of EC subset identity marker genes: Ramp3 (metaphyseal ECs, mpECs), Gja4 (arterial ECs, aEC), Fmo (arteriole-like ECs, alECs) and Stab2 (bone marrow ECs, bm) (f). g, h. Volcano plot of differentially expressed genes (frac. expressed > 0.2 in either condition) between control and mutant condition across all ECs (blue color: log2FC >=1,p.adj <=0.001,) (g). Visualisation of selected known Hippo pathway target genes: Ccn1 (Cyr61), Ccn2 (Ctgf), Angpt2 and Thbs1 in Lats2iΔEC mutant and control (h). [file 44161_2024_508_Fig16_ESM.jpg]

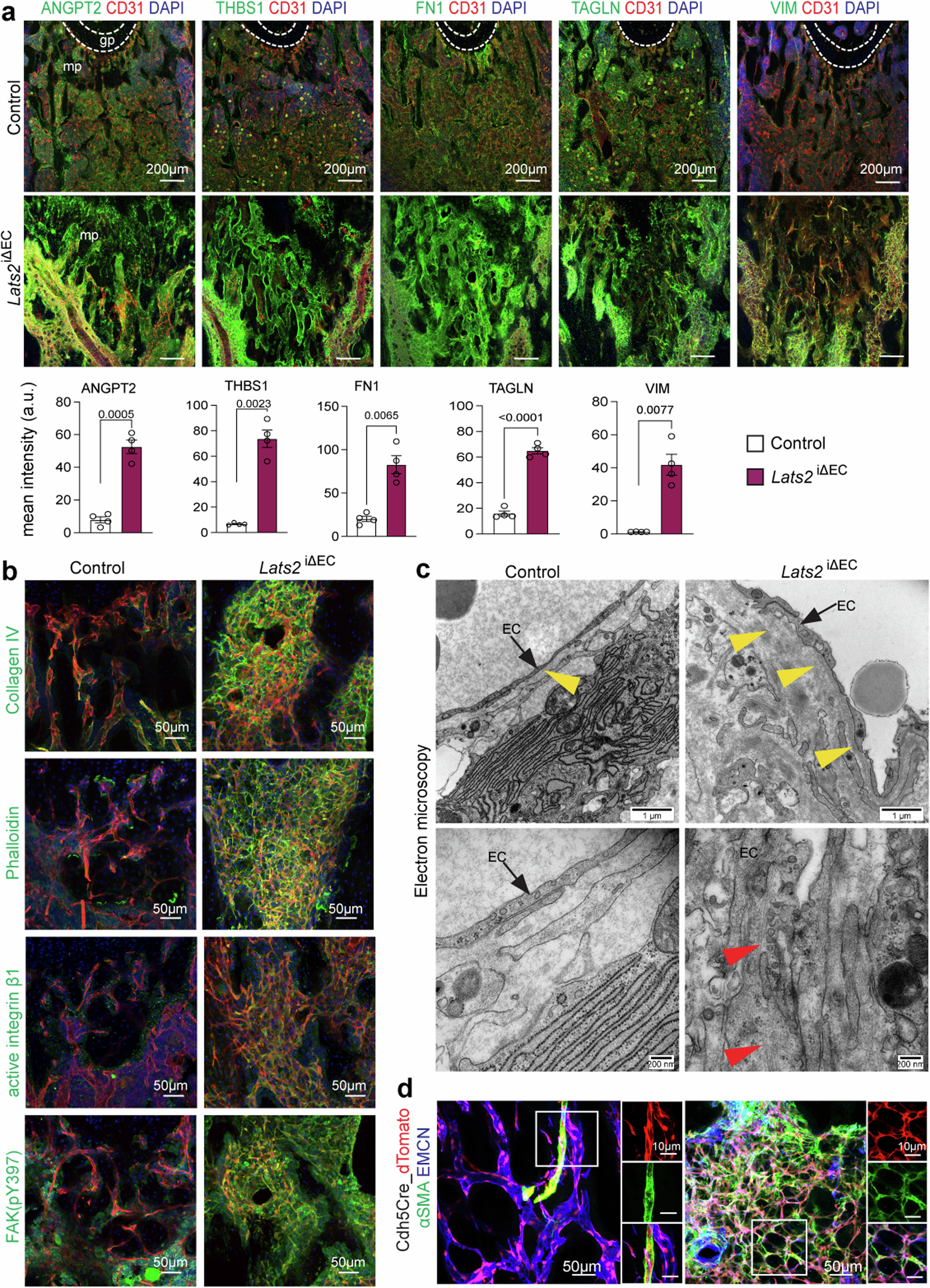

Supplement: EndMT in Lats2iΔEC bone endothelium. — a. Confocal images showing strongly elevated Angiopoietin 2 (ANGPT2), Thrombospondin 1 (THBS1), Fibronectin 1 (FN1), Transgelin (TAGLN), and Vimentin (VIM) expression in Lats2iΔEC bone sections. (n = 4). Data in graphs are presented as mean ± SEM. P values, statistical analysis performed using the Mann–Whitney test (two-tailed)). b. Confocal images showing increased staining for collagen IV (COL IV), the actin cytoskeleton (with Phalloidin), active-integrin β1 and phosphorylated focal adhesion kinase (FAK pY397) in Lats2iΔEC mutant relative to control. c. Transmission electron micrographs showing increased subendothelial basement membrane and accumulation of ECM (yellow arrowheads) in Lats2iΔEC femur. Mutant ECs are rich in actin filaments (red arrowheads). d. Confocal images of Lats2iΔEC mutant in R26-dTomato reporter background showing αSMA staining in dTomato+ ECs. In control samples, αSMA signal is confined to SMCs covering arteries. n = independent biological samples. Source data [file 44161_2024_508_Fig17_ESM.jpg]

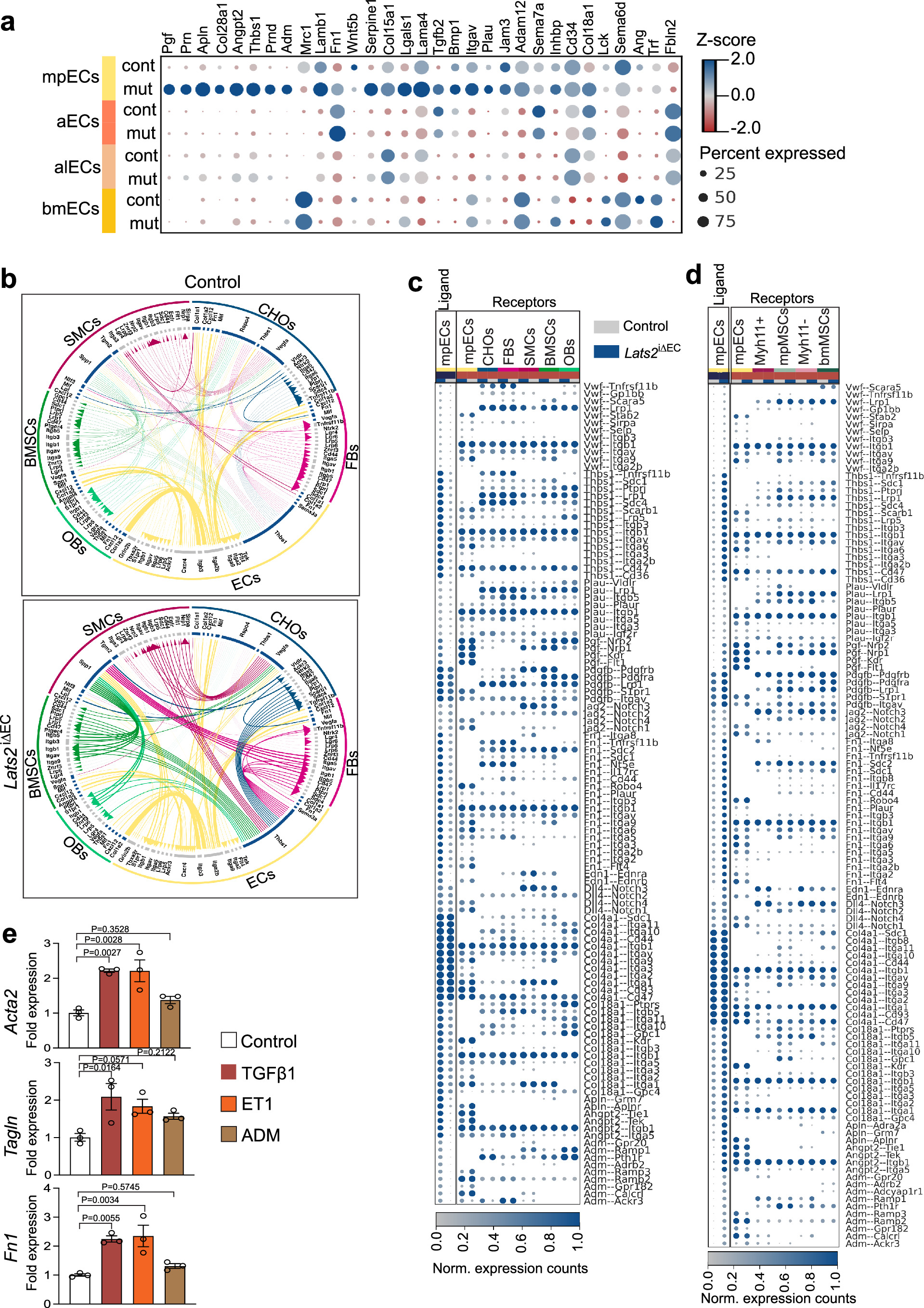

Supplement: Angiocrine signals and EC-BMSC cross-talk. — a. Ligands showing on average significantly (log2FC >=1,p.adj <=0.05, frac. expressed >=0.1) larger counts in mutant mpECs than in any other EC subidentity and are significantly elevated (see Methods) relative to at least one other EC subpopulation. b. Selected ligand-receptor parings of main bone stromal cells, which are increased in Lats2iΔEC mutants. c. Mean normalized expression counts of selected, significant ligand-receptor interactions (p.adj of LRI score in either condition and LRIDiff Scores <= 0.05, |LRIDiff| >= 1) in mpECS and all other major celltypes. Ligand (left, dark blue bar) and receptor (right, red bar). d. Mean normalized expression counts of selected, significant ligand-receptor interactions (p.adj of LRI score in either condition and LRIDiff Scores <= 0.05, LRIDiff >= 1) in mpECS, mpMSCs and SMCs. Ligand (left, dark blue bar) and receptor (right, red bar). e. Quantitative PCR (qPCR) shows expression of myofibroblasts markers Acta2, Tagln and Fn1 in cultured BMSCs upon treatment with recombinant TGFβ1, endothelin 1 (ET1) or adrenomedullin (ADM). (n = 3). Data in graphs are presented as mean ± SEM. P values, statistical analysis performed using the Mann–Whitney test (two-tailed)). n = independent biological samples. Source data [file 44161_2024_508_Fig18_ESM.jpg]

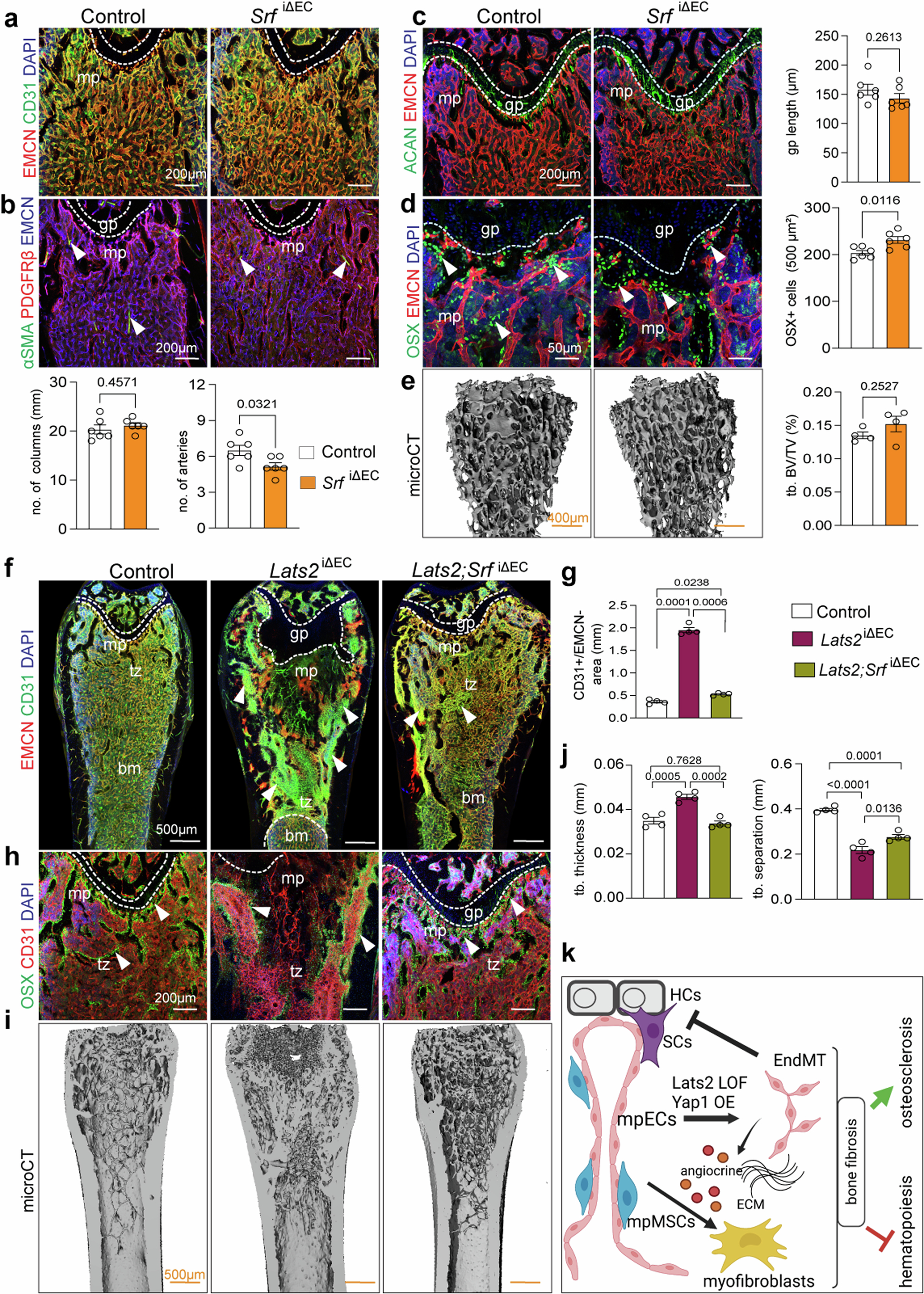

Supplement: Lats2 and SRF control EC behavior. — a, b. Representative confocal images of control and SrfiΔEC femur show type H (EMCNhigh; CD31high) capillaries, Nuclei, DAPI (blue) (a) as well as PDGFRβ+ cells (red) and αSMA+ arterial SMCs (green) and EMCN (blue) in the femoral metaphysis (b). Graphs in (b) show quantification of vessel columns and arteries (n = 6). Data presented as mean ± SEM. P values, Mann–Whitney test (two-tailed). c-e. Confocal images showing ACAN+ (green) chondrocytes in femoral growth plate (gp) (c) and OSX+ cells (green) (d). µCT images of trabecular bone of SrfiΔEC mutant and control(e) and quantitation of trabecular (tb) volume (BV/TV, bone volume/total volume) tb number and gp length (n = 6). Data presented as mean ± SEM. P values, Mann–Whitney test (two-tailed). f, g. Longitudinal tile scan confocal images showing EMCN+ (red) CD31+ (green) vessels in control, Lats2iΔEC and Lats2 SrfiΔEC compound mutant femur (f). Metaphysis (mp), growth plate (gp, dashed lines) and bone marrow (bm) are indicated. Quantification shows increase in CD31+ EMCN- area in Lats2iΔEC metaphysis and rescue by inactivation of Srf (g) (n = 4). Data presented as mean ± SEM. P values, Tukey multiple comparison test (one-way Anova). h. Representative images showing OSX+ cells in control, Lats2iΔEC, and Lats2 SrfiΔEC compound mutant femur. i, j. Representative μCT images of femoral trabecular bone (i). Quantitative analysis of trabecular bone (tb) thickness and separation (j) (n = 4). Data presented as mean ± SEM. P values, Tukey multiple comparison test (one-way Anova). k. Proposed model of YAP1/TAZ-induced endothelial-to-mesenchymal transition (EndMT) as a driver of myelofibrosis and osteosclerosis. n = independent biological samples. Created with BioRender.com. Source data [file 44161_2024_508_Fig19_ESM.jpg]
