# Supplementary material for: Endothelial LATS2 is a suppressor of bone marrow fibrosis
Source: Nat Cardiovasc Res. 2024 Jul 29;3(8):951–69. doi: 10.1038/s44161-024-00508-x (PMC11324521; doi:10.1038/s44161-024-00508-x)
Supplement: Supplementary file 11 — Supplementary Fig. 1 and Supplementary Tables 1 and 2. [file 44161_2024_508_MOESM1_ESM.pdf]

nature cardiovascular research

Supplementary information https://doi.org/10.1038/s44161-024-00508-x

Endothelial LATS2 is a suppressor of bone marrow fibrosis

In the format provided by the authors and unedited

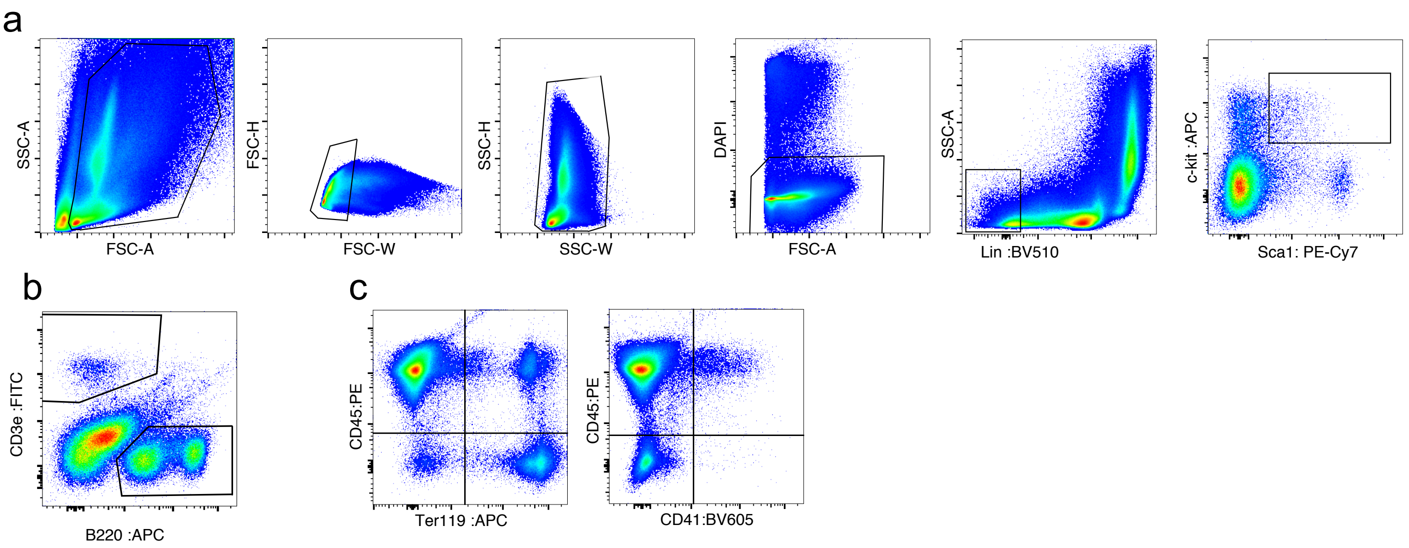

Supplementary Figure 1

Representative gating stragegy for the FACS LSK (a), CD3e+ and B220+ cells (b), CD45+, Ter119+, and CD41+ cells (c).

Markers and settings for scRNA-seq analysis

Supplementary Table 1.

Cell Identity	Marker Genes used
Endothelial Cells	Cdh5+, Emcn+, Vwf+, Prox1+
- mp-ECs	Nox4+ (specific to cKO), Ramp3+, Aplnr+
- bm-ECs	Stab2+
- a-ECs	Gja4+
- al-ECs	Vwf+, Fmo2+, Tagln+, Aplnr+
BMSCs	Esm1+, Adipoq+, Kitl+, Pdgfrb+, Wif1+
- mp-MSC (Osteo-CAR)	Postn+, Wif1+
- bm-MSC (Adipo-CAR)	Adipoq+, Esm1+,
Osteoblastic Cells	Bglap2+, Bglap3+, Car3+, Phex+
Fibroblasts	Pdgfra+, Igfbp6+, Slurp1+, S100a4+, Sod3+
SMCs	Pdgfrb+, Rgs5+, Myh11+, Acta2+
- Myh11+ SMCs	Myh11+
- Myh11- SMCs	Myh11-
Chondrocytes	Prkg2+, Col9a1+, Sox9+, Acan+, Prg4+, Slurp1-
- Fibroblasts	Igfbp6+, Prg4+, Thbs4+, Slurp1+
- Chondrocyte Progenitors	Prg4+, Acan+, Slurp1-
- Columnar Chondrocytes	Sfrp5+, Acan+, Thbs4+
- Pre-hypertrophic Chondrocytes	Acan+, Ihh-, Vegfa-
- Hypertrophic Chondrocytes	Acan+, Ihh+ (early), Vegfa+ (late)
Proliferating Cells	Mki67+, Top2a+

Supplementary Table 2.

Cell Identities Subset	Highly Variable Genes	Principle Components calculated	Leiden clustering resolution	Second Passthrough?
Endothelial Cells	3000	30	0.5	x
BMSC and Osteoblastic Cells	3000	30	0.1	x
Chondrocytes	4000	40	0.8	
SMCs	3000	20	0.2	
Merging of all above Subsets	3000	50	- (annotations from subsetting kept)	
Merging of MSC & SMC Subsets	3000	40	- (annotations from subsetting kept)	
