# Supplementary material for: Endothelial LATS2 is a suppressor of bone marrow fibrosis
Source: Nat Cardiovasc Res. 2024 Jul 29;3(8):951–69. doi: 10.1038/s44161-024-00508-x (PMC11324521; doi:10.1038/s44161-024-00508-x)
Supplement: Supplementary file 12 — Reporting Summary [file 44161_2024_508_MOESM2_ESM.pdf]

nature portfolio

Corresponding author(s): Ralf H. Adams, Kishor K. Sivaraj and Rebekka K. Schneider

Last updated by author(s): Jun 5, 2024

Reporting Summary

Nature Portfolio wishes to improve the reproducibility of the work that we publish. This form provides structure for consistency and transparency in reporting. For further information on Nature Portfolio policies, see our Editorial Policies and the Editorial Policy Checklist.

Statistics

For all statistical analyses, confirm that the following items are present in the figure legend, table legend, main text, or Methods section.

n/a	Confirmed	
☐	☒	The exact sample size (*n*) for each experimental group/condition, given as a discrete number and unit of measurement
☐	☒	A statement on whether measurements were taken from distinct samples or whether the same sample was measured repeatedly
☐	☒	The statistical test(s) used AND whether they are one- or two-sided<br>*Only common tests should be described solely by name; describe more complex techniques in the Methods section.*
☐	☒	A description of all covariates tested
☐	☒	A description of any assumptions or corrections, such as tests of normality and adjustment for multiple comparisons
☐	☒	A full description of the statistical parameters including central tendency (e.g. means) or other basic estimates (e.g. regression coefficient) AND variation (e.g. standard deviation) or associated estimates of uncertainty (e.g. confidence intervals)
☐	☒	For null hypothesis testing, the test statistic (e.g. *F*, *t*, *r*) with confidence intervals, effect sizes, degrees of freedom and *P* value noted<br>*Give P values as exact values whenever suitable.*
☒	☐	For Bayesian analysis, information on the choice of priors and Markov chain Monte Carlo settings
☒	☐	For hierarchical and complex designs, identification of the appropriate level for tests and full reporting of outcomes
☒	☐	Estimates of effect sizes (e.g. Cohen's *d*, Pearson's *r*), indicating how they were calculated

Our web collection on statistics for biologists contains articles on many of the points above.

Software and code

Policy information about availability of computer code

Data collection	RNA sequencing by NestSeq500, MiSeq (Illumina); Confocal immunofluorescence image were collected from leica TCS-SP8; Cell sorting and analyzed by FACS Aria II cell sorter (BD Bioscience); GraphPad Prism (version 9). Adobe Photoshop 2021 and Adobe illustrator 2021.
Data analysis	Data analysis and statistical tests were done using GraphPad Prism (Version 9). Confocal images were analyzed with Volocity 6.3 (Quorum Techonology) or imageJ(Version:2.0.0-re-69/1.52p). For FACS data analysis, we used FACSDiva v8.02 and flowjo.v10.<br><br>Single cell RNA sequencing data analysis<br>Raw sequencing data was converted to fast format using bcl2fastq (version 2.20.0.422). Sequencing reads were processed with cutadapt (version 3.5) and aligned and counted with STAR (version 2.7.l0a). All further analyses were conducted with custom code and using existing modules, mainly matplotlib (version 3.5.3), Ann Data (version 0.8.0), scanpy (version 1.9.1), harmonypy (0.0.9), scran (version 1.24.1), scDblFinder (version 1.11.4), DESeq2 (version 1.36), igraph (version 0.10.4), sklearn (version 1.1.2), statsmodels (version 0.13.1), scipy (version 1.9.1), milopy (version 0.1.0) and slingshot (2.6.0). The major programming languages used were R (version 4.2.1) and python (version 3.8.10). Also the iRegulon plugin to Cytoscape was used (version 3.9.1). All code used to produce the computational analysis results and figures in this publication are available at https://gitlab.gwdg.de/pmajev/sivaraj_majev_lats2_ecs (10.5281/zenodo.11456485).

For manuscripts utilizing custom algorithms or software that are central to the research but not yet described in published literature, software must be made available to editors and reviewers. We strongly encourage code deposition in a community repository (e.g. GitHub). See the Nature Portfolio guidelines for submitting code & software for further information.

Data

Policy information about availability of data

All manuscripts must include a data availability statement. This statement should provide the following information, where applicable:

- Accession codes, unique identifiers, or web links for publicly available datasets
- A description of any restrictions on data availability
- For clinical datasets or third party data, please ensure that the statement adheres to our policy

Data availability
The scRNA-seq data generated in this study have been deposited in the gene expression omnibus (GEO) database under the accession number GSE225429 (https://www.ncbi.nlm.nih.gov/geo/query/acc.cgi?acc=GSE225429). The mouse reference genome GRCm39 with Gencode M26 anotation (https://www.gencodegenes.org/mouse/release_M26.html) was used for mapping the reads to in this study. All other relevant data supporting the key findings of this study are available within the article and its Supplementary Information files or from the corresponding author upon reasonable request.

Code availability
All code used to produce the computational analysis results and figures in this publication are available at https://gitlab.gwdg.de/pmajev/sivaraj_majev_lats2_ecs. The assigned DOI is 10.5281/zenodo.11456485 (https://doi.org/10.5281/zenodo.11456485).

Human research participants

Policy information about studies involving human research participants and Sex and Gender in Research.

Reporting on sex and gender	N/A
Population characteristics	N/A
Recruitment	N/A
Ethics oversight	N/A

Note that full information on the approval of the study protocol must also be provided in the manuscript.

Field-specific reporting

Please select the one below that is the best fit for your research. If you are not sure, read the appropriate sections before making your selection.

☒ Life sciences ☐ Behavioural & social sciences ☐ Ecological, evolutionary & environmental sciences

For a reference copy of the document with all sections, see nature.com/documents/nr-reporting-summary-flat.pdf

Life sciences study design

All studies must disclose on these points even when the disclosure is negative.

Sample size	No specific statistical methods were used to predetermine sample size. Sample size were chosen based on previous experience. (ref. kusumbe et al. Nature 2014; Ramasamy et al., Nature 2014; Sivaraj et al., Elife 2020; Sivaraj et al., Cell Report 2021).
Data exclusions	Cells are excluded in the single cell RNA-seq data. Cells with less than 1000 UMI or 800 features (>0 counts per feature) detected, were discarded. Similarly, only cells with less than 15% of counts stemming from mitotic genes or 5% from hemoglobin genes were retained. By these strict thresholds mostly remaining cells of the haematopoietic lineage and low quality cells were removed. Repeating the analysis without these cut-offs revealed no additional desired cell types. Only features present with more than 10 counts total from at least 10 cells were kept.
Replication	scRNA-seq experiments were performed in duplicates and other all experiments were repeated at least three times and performed independently to ensure reproducibility. All the attempts of replication experiments were successful.
Randomization	No formal method of randomization was used. All experiments involving wildtype mice were performaed on inbred C57Bl6 strain with male mice of same age group. For mutant studies we used both male and female of same age group, and phenotype were always compared between same age and sex of animals. For Fibrosis model we used age and sex matched mice.
Blinding	When appropriate, data analysis was done in a blinded manner before being unblinded once the analyses were finished.

Reporting for specific materials, systems and methods

We require information from authors about some types of materials, experimental systems and methods used in many studies. Here, indicate whether each material, system or method listed is relevant to your study. If you are not sure if a list item applies to your research, read the appropriate section before selecting a response.

Materials & experimental systems

n/a	Involved in the study
☐	☒ Antibodies
☒	☐ Eukaryotic cell lines
☒	☐ Palaeontology and archaeology
☐	☒ Animals and other organisms
☒	☐ Clinical data
☒	☐ Dual use research of concern

Methods

n/a	Involved in the study
☒	☐ ChIP-seq
☐	☒ Flow cytometry
☒	☐ MRI-based neuroimaging

Antibodies

Antibodies used

All antibody details (clone and manufacturer) are included in methods and also listed below:
Antibody Manufacturer Catalog number Concentration
Rat monoclonal anti-Endomucin (V.7C7) (Santa Cruz, Cat# sc-65495, 1:100 dilution),
Goat polyclonal anti-CD31 (R&D, Cat# AF3628, 1:100 dilution),
Rabbit-polyclonal anti-Fabp5 (Lifespan Bioscience, Cat#C312991, 1:100 dilution),
Goat anti-PDGFRβ (R&D, Cat# AF1042, 1:100 dilution),
Mouse monoclonal alpha-smooth muscle actin-Cy3 (Sigma, Cat#C6198, 1:200 dilution),
Mouse monoclonal alpha-smooth muscle actin-eFluor660 (eBioscience, Cat#50-9760-82, 1:200 dilution),
Rabbit monoclonal anti-Yap1/Taz (D24E4, Cell signaling, Cat#8418, 1:100 dilution),
Rabbit monoclonal anti-Vimentin (D21H3, Cell signaling, Cat#5741, 1:100 dilution),
Rabbit polyclonal anti-Tagln (Sm22a, Abcam, Cat#ab14106, 1:100 dilution),
Rabbit polyclonal anti-Fibronectin (Fn1, Sigma, Cat#F3648, 1:100 dilution),
Rabbit polyclonal anti-Thrombospondin 1 (Thbs1, Abcam, Cat#ab85762, 1:100 dilution),
Rabbit polyclonal anti-Angpt2 (Abcam, Car# ab8452, 1:100 dilution),
Rabbit polyclonal anti-Cav1 (Cell signaling, Cat#3238, 1:100 dilution),
Rabbit polyclonal anti-Col-I (Millipore, Cat#AB765P, 1:100 dilution),
Rabbit polyclonal anti-Col-X (Abcam, Cat#ab58632, 1:100 dilution),
Rabbit polyclonal anti-LATS2 (GeneTex, Cat#GTX87529, 1:50 dilution),
Rabbit polyclonal anti-Col4 (AbD Serotec, Cat# 2150-1470, 1:100 dilution),
Rabbit-polyclonal anti-Osterix (Abcam, Cat#ab22552, 1:300 dilution),
Roat polyclonal anti-Mmp9 (R&D, Cat#AF909, 1:200 dilution),
Rabbit polyclonal anti-Aggrecan (Milipore, Cat#AB1031, 1:100 dilution),
Rabbit anti-vATPaseB1/B2 (Abcam, Cat#200839, 1:100 dilution),
Rat anti-mouse Ter119 (BD Bioscience, Cat#553673, 1:200 dilution),
Rat anti-mouse B220 (BD Bioscience, Cat#553090, 1:200 dilution),
Rat-monoclonal anti-active Itgb1(BD Bioscience, Cat#553715, 1:50 dilution),
Rabbit polyclonal anti-FAK (pY397) (ThermoFisher, Cat#44-624G, 1:50 dilution),
Rabbit monoclonal anti-Runx2 (Abcam, Cat#ab192256, 1:200 dilution),
Rat monoclonal anti-Srf (from Alfred Nordheim, 1:50 dilution),
lineage cell detection cocktail-biotin (Miltenyi Biotec, Cat#130-092-613, 1:100 dilution),
Rat monoclonal anti-Ly6A-PE-cy7 (BD Pharmingen, clone D7, Cat# 558162, 1:100 dilution),
Rat monoclonal anti-CD117-APC (BD Pharmingen, clone 2B8, Cat# 553356, 1:100 dilution),
Hamster monoclonal anti-CD3e-FITC eBioscience, clone 145-2C11, Cat# 11-0031, 1:100 dilution),
Rat monoclonal anti-B220-APC (Invitrogen, RA3-6B2, Cat# RM2605, 1:100 dilution),
Rat monoclonal anti-CD11b-APC (Biolegend, M1/70, Cat# 101212, 1:100 dilution),
Rat monoclonal anti-Ly-6G(Gr-1)-BV605 (Biolegend, RB6-8C5, Cat# 108440, 1:100 dilution),
Rat monoclonal anti-CD45-PE (BD Pharmingen, 30-F11, Cat# 553081, 1:100 dilution),
Rat monoclonal anti-TER-119-APC (eBioscience, Cat# 17-5921, 1:100 dilution),
Rat monoclonal anti-CD41-BV605 (Biolegend, MWReg30, Cat# 133921, 1:100 dilution)
Alexa Fluor 488 (Thermo Fischer Scientific, Cat#A21208, 1:100 dilution)
Alexa Fluor 546 (Thermo Fischer Scientific, Cat#A11056, 1:100 dilution)
Alexa Fluor 594 (Thermo Fischer Scientific, Cat#A21209, 1:100 dilution)
Alexa Fluor 647 (Thermo Fischer Scientific, Cat#A31573 or Cat#A21447, 1:100 dilution)
Phalloidin -488 (Invitrogen, Cat# A12379, 1:100 dilution)

Validation

All antibodies used in the study have been commercially available and previously used by our group (ref. kusumbe et al. Nature 2014; Ramasamy et al., Nature 2014; Sivaraj et al., Elife 2020; Sivaraj et al., Cell Report 2021). The complete information and all validation information for each Ab as well as previous publications that have used each Ab can be found on the manufacturer's website.

Animals and other research organisms

Policy information about studies involving animals; ARRIVE guidelines recommended for reporting animal research, and Sex and Gender in Research

Laboratory animals	All animals used in this study are Mus musculus species, C57/BL6 background strain independent of genotype. All mice were maintained in individually ventilated cages (IVC), with constant access to food and water under a 12 h light and 12 h dark cycle regime. Air flow, temperature (21–22 °C) and humidity (55–60%) were controlled by an air management system. Animals were checked daily and maintained in specific pathogen-free (SPF) conditions. Sufficient nesting material and environmental enrichment was provided.<br><br>Transgenic mice were generated from our laboratory, Cdh5(PAC)-CreERT2, YapKI mice; Yap1/Taz flox/flox mice from Helen McNeill Laboratory; Lats2 flox/flox from Dae-Sik Lim Laboratory, KAIST, Korea; Srf flox/flox mice from Alfred Nordheim; and follwoing transgenic mice Rosa26-mTG were purchased from Jackson Laboratory. 12-week old male and female trangenic mice were used for most of the experiments.<br>For mutant experiment , mice were bred to Cdh5(PAC)Cre -ERT2 to generate inducible mouse model. Cre negative were used as litter mate control. Most of the experiments were performed at 12 weeks.<br><br>Fibrosis ThPO mouse experiment were performed on 10-12 weeks-old mice and analyzed in 8 - 10 weeks later.
Wild animals	No wild animals were used in the study.
Reporting on sex	Male and female mice were used for most of the experiments.
Field-collected samples	No field collected samples were used.
Ethics oversight	All animal experiments were performed according to the international guidelines and laws, approved by local animal ethical committee University of Muenster and Max Planck Institute for Molecular Biomedicine with premissions granted by the Landesamt fur Natur, Umwelt und Verbraucherschutz (LANUV) of North Rhine-Westphalia. Germany.<br><br>ThPO and MPLW515L bone fibrosis studies were conducted according to protocols approved by the Central Animal Committee (Centrale Commissie Dierproeven [CCD], Netherlands) in accordance with legislation in The Netherlands.

Note that full information on the approval of the study protocol must also be provided in the manuscript.

Flow Cytometry

Plots

Confirm that:

☒ The axis labels state the marker and fluorochrome used (e.g. CD4-FITC).

☒ The axis scales are clearly visible. Include numbers along axes only for bottom left plot of group (a 'group' is an analysis of identical markers).

☒ All plots are contour plots with outliers or pseudocolor plots.

☒ A numerical value for number of cells or percentage (with statistics) is provided.

Methodology

Sample preparation	Bone marrow (BM) isolation was performed using crushing methodology, followed by MACS beads and quadroMACS (LS columns) were used for lineage depletion or bone marrow stromal cells enrichment. We used femur and tibia are seperated by surgical dissection and bone marrow were collected. Bones were dissected into small size and digested with Collagenase for 30 min at 37° C. Cells were washed with 1%BSA and filtered to obtained a single cell suspension. The detailed BM isolation protocol is included in the methods section.
Instrument	FACS Aria II cell sorter (BD Bioscience)
Software	FACSDiva (BD Bioscience) was used for sorting and analysis.
Cell population abundance	Sorted cell quality were analysed by confocal microscopy.
Gating strategy	Single viable cells were gated initally forward and side scatter.

☒ Tick this box to confirm that a figure exemplifying the gating strategy is provided in the Supplementary Information.
